# Straw/Nano-Additive Hybrids as Functional Fillers for Natural Rubber Biocomposites

**DOI:** 10.3390/ma14020321

**Published:** 2021-01-09

**Authors:** Justyna Miedzianowska, Marcin Masłowski, Przemysław Rybiński, Krzysztof Strzelec

**Affiliations:** 1 Institute of Polymer & Dye Technology, Lodz University of Technology, Stefanowskiego 12/16, 90-924 Lodz, Poland; marcin.maslowski@p.lodz.pl (M.M.); krzysztof.strzelec@p.lodz.pl (K.S.); 2 Institute of Chemistry, Jan Kochanowski University, Żeromskiego 5, 25-369 Kielce, Poland; przemyslaw.rybinski@ujk.edu.pl

**Keywords:** straw, hybrid fillers, natural rubber, biocomposites, functional properties, flammability

## Abstract

Currently, up to 215 million metric tons of harvestable straw are available in Europe, 50% of the crops come from wheat, 25% from barley and 25% from maize. More than half of the production remains undeveloped. The overproduction of straw in the world means that the current methods of its management are insufficient. The article describes the production method and characterization of natural rubber biocomposites containing cereal straw powder modified with functional nano-additives in the form of carbon black, silica and halloysite nanotubes. The use of cereal straw in the elastomer matrix should contribute to obtaining a product with good mechanical properties while ensuring a low cost of the composite. In turn, the application of the mechanical modification process will allow the combination of specific properties of raw materials to obtain new, advanced elastomeric materials. As part of the work, hybrid fillers based on mechanically modified cereal straw were produced. The impact of hybrid fillers on mechanical, rheometric and damping properties was assessed. The flammability and susceptibility of the obtained biocomposites to aging processes were determined. The use of hybrid fillers based on mechanically modified straw allowed us to obtain a higher cross-linking density of vulcanizates (even up to 40% compared to the reference sample), and thus higher values of the rheometric moment during the vulcanization process of rubber mixtures (from approx. 10% (10 phr of filler) up to 50% (30 phr of filler) in relation to the unfilled system) and higher hardness of vulcanizates (by about 30–70%). The curing time of the blends was slightly longer, but the obtained composites were characterized by significantly higher tensile strength. The use of fillers in the elastomer matrix increased the modulus at 100, 200 and 300% and the elongation at break. Moreover, greater resistance of vulcanizates to the combustion process was confirmed.

## 1. Introduction

In recent years, discussions on natural resource prevention and recycling have led to a special interest in biomaterials. Due to growing environmental awareness, limited petrochemical resources, government regulations and changes in company policy, the use of traditional composites based on epoxy resins, unsaturated polyesters, glass [1], carbon [2] or aramid [3] fiber-reinforced phenols is under critical scrutiny. Advances in the use of natural fibers, the development of genetic engineering and composite technologies have created opportunities to improve materials from renewable resources while pursuing the idea of sustainable development [4,5,6,7].

A primary factor in the correct approach to biocomposite production is knowledge of the structure and properties and functions of the matrix material, as well as how it connects and interacts with other additives in biocomposite formation [8]. The shape, surface appearance and durability are determined by the composite matrix. Fibrous reinforcement provides macroscopic stiffness and strength [9]. The polymer market is dominated by plastics based on non-renewable crude oil resources. World literature reports in the field of materials containing plant bio-additives usually include composites based on thermoplastics and thermosetting [10]. The number of publications available on the use of natural fillers in elastomeric materials is limited. Natural rubber (NR) may be the polymeric medium of naturally derived fiber-filled composites. As an elastomer, it is a material that shows a high degree of deformation at low stresses in terms of operating and storage temperatures [11]. The most important properties of NR are: good calenderability and extrudability, high tensile strength of unfilled cross-linked rubber, ease of conventional cross-linking and non-polarity resulting in relatively good resistance to polar solvents [12].

The second and most important factor determining the properties of biocomposites is the selection of appropriate natural fibers as a reinforcement of biocomposites. Their low density and cost, stiffness and relatively high strength make natural fibers materials that strengthen the structure of natural fiber polymer composites (NFPCs). The properties of natural fibers may differ significantly from conventional carbon, glass or aramid fibers [13]. Their characteristics depend on the age of the plant [14], the place of origin [15] or the initial preparation [16]. These factors also determine the chemical composition of natural fibers [17]. Their classification according to their origin includes several groups, such as fibers from seeds, stems, leaves, fruit, wood, grasses and straw [18].

Straw consists of the leaves and stems of mature, dried crops: rape, poppy, flax, cereals and legumes [19]. The dry matter content in straw is approx. 85% and its main component is crude fiber. According to [20], up 215 million metric tons of harvestable straw are available in Europe; of this, 50% is from wheat, 25% from barley and 25% from corn. The main areas of utilization of this waste from seed production are as bedding material, fertilizer, feed ingredients or fuel in the energy sector [21,22]. Traditionally, large amounts of straw are burned in the fields and the remains are plowed and used as fertilizer. Obviously, such use has a negative impact on the natural environment through the emission of harmful substances and poses a threat to living organisms. Part of the overproduction of straw is managed as animal feed or bedding or as a substrate for mushroom plantations. Moreover, straw can be used to generate energy through the production of bioethanol, biogas and bio-oil, as well as by direct combustion. It is also known for containing natural, environmental absorbents for pollution control as well as construction materials [23,24,25]. The methods of straw management are characterized by certain limitations, hence, there is still a need to develop new methods and technologies for its use. The straw surplus can be successfully used in polymer composites [26,27]. Composites containing various types of plant fillers are produced on an industrial scale and continuous research results in the continuous expansion of the area of their application. The use of straw in the elastomer matrix contributes to obtaining a product with good mechanical properties while ensuring the low cost of the composite because this raw material is characterized by easy availability and low unit price [28,29,30,31]. Individual straw fibers have a much lower tensile strength and lower compressive strength compared to other fibers that are added to polymers. A limited number of studies have reported the use of straw fibers for composite production [32]. For this reason, it is important to know and examine another form of straw, in this case the powder.

Although the chemical composition of natural fibers is very diverse, almost all of them are hydrophilic [33]. The content of highly crystalline, linear cellulose reduces the hydrophilicity of the fibers, however, the free hydroxyl groups available on their surface are still able to absorb water. Most of the polymer matrices used in NFPCs are hydrophobic, which leads to the problem of poor wettability of the fibers [34,35]. As a result, voids are created in the composite and the effective transmission of stresses is disturbed. Composites exposed to weather conditions may show dimensional instability, which is directly related to the swelling of fibers due to water absorption [36]. The stresses generated in this way can damage the composite. Moreover, high moisture content increases biodegradability. Several concepts have emerged to overcome these problems. The main goal is to create a bond between the matrix and the reinforcement, which would improve the compatibility of the main components of the composite with each other through, for example, physical modification. Physical modifications, such as the use of low-temperature plasma, corona discharge, stretching, calendering, thermal or mechanical treatment, change the structural and surface properties, thus improving the mechanical bond of the fibers with the matrix without significantly affecting the chemical composition. The main advantage of physical modifications is the environmental aspect because in the case of chemical treatments, large amounts of solvents are used [37,38].

Hybridization is a modification method that combines individual, seemingly different properties of additives. This is a particularly fascinating technique used in fillers for polymer composites. It consists in creating new materials from substrates that appear physically and chemically different. As a result, this creates a synergistic effect, a combination of properties affecting the entire material at the same time.

Recent years have seen rapid development in the manufacture of a new group of polymeric materials, namely polymer nanocomposites [39,40,41]. Reducing the particle size of substances introduced into the polymer matrix from micrometers to nanometers gives the possibility to obtain composites with better, and often completely new, properties [42,43,44].

- Halloysite nanotubes were first described by Berthier in 1826 as a dioctahedral 1:1 clay mineral of the kaolin group. The multilayer tubular structure of halloysite results from the wrapping of the 1:1 clay mineral layers under favorable geological conditions, driven by a mismatch in the oxygen-sharing tetrahedral and octahedral sheets in the 1:1 layer. Halloysite has a structural formula of Al_2_(OH)_4_Si_2_O_5_·nH_2_O [45]. The length of halloysite tubes varies from 1 to 15 micrometers, while their diameter ranges from 10 to 150 nanometers. Thus, they are characterized by a large geometric coefficient of shape, which can have a positive effect on the mechanical properties of the polymer. Besides, halloysite exhibits a less hydrophilic character compared to other aluminosilicates, which in turn should facilitate its miscibility with nonpolar polymers, such as natural rubber.
- Nanoscale carbon black is one of the widely used nanofillers because of its abundant source, low density, permanent conductivity and low cost [46]. The primary particle size indicated the reinforcement effect in composites. Carbon black with a smaller particle size reinforces the composite more effectively than a larger particle size [47]. Carbon black is also a radical deactivator because on its surface there are unpaired electrons. Its presence inhibits radical reactions while increasing the likelihood of the recombination of primary macroradicals by prolonging their residence time in the cage [48]. Carbon particles located in the boundary layer additionally act as sorbents of volatile elastomer decomposition products [49].
- Nanosilica is silicon dioxide SiO_2_. The silica molecule is in the form of a tetrahedron with a silicon atom in the center, surrounded by four oxygen atoms in the corners of this geometrical structure [50]. Chemically, the surface of silica is almost passive. Silica has a well-developed specific surface area of several dozen to several hundred m^2^/g and a porous structure [51], which promotes the strengthening of polymer composites. The structure of nanosilica shows a three-dimensional network [52]. Silanol and siloxane groups are created on the silica surface, leading to the hydrophilic nature of the particles [53]. The surfaces of the silica are typically terminated with three silanol types: free or isolated silanols, hydrogen-bonded or vicinal silanols and geminal silanols [54].

The work aimed to produce natural rubber biocomposites containing grain straw powder modified with nanotubes. Composites containing various types of plant fillers are already produced on an industrial scale and continuous research results in the continuous expansion of the area of their application. The use of cereal straw in the elastomer matrix should contribute to obtaining a product with good mechanical properties while ensuring a low cost of the composite, as this raw material is characterized by easy availability and a low unit price. Straw is considered a common and cheap raw material, the purchase price is on average EUR 25 per ton, but many farmers are willing to give this valuable material for free due to the large overproduction and the lack of generally available management methods. Additionally, to eliminate the negative properties of cereal straw as a bio-additive of natural rubber, modifiers were used: carbon black, silica and halloysite. The mechanical modification should allow for the production of advanced materials that will combine the specific properties of the components used.

## 2. Materials and Methods

### 2.1. Materials

Three different types of straw-based fillers were used in the study. The materials were prepared by hybridizing wheat straw (local farms from Poland) with nano-additives. Silica, halloysite and carbon black were proposed as functional agents:

- Industrially precipitated silica ULTRASIL^®^ VN 3 (Evonik Industries AG, Essen, Germany) with a specific surface area of 180 m^2^/g;
- The carbon black, HAF N-339 (Konimpex, Konin, Poland) with a specific surface area of 91 m^2^/g;
- The halloysite was applied in the form of nanotubes with a specific surface of 64 m^2^/g, the material was supplied by Sigma-Aldrich (Saint Louis, MO, USA).

Natural rubber (NR) (Torimex-Chemicals Ltd. Sp. z o.o, Lodz, Poland) in the form of ribbed smoked sheets (RSS 1) was used as the elastomer matrix. The elastomeric composites were cross-linked with the use of a sulfur system containing [55]: 2 parts per hundred rubber (phr) sulfur (S) (Siarkopol, Tarnobrzeg, Poland), 2 phr mercatobenzothiazole (MBT) (Saint Louis, MO, USA), 1 phr stearic acid (SA) (Avantor Performance Materials, Gliwice Poland) and 5 phr zinc oxide (ZnO) (Huta Bedzin, Poland). Compositions of elastomer mixtures are presented in Table 1.

The reference sample was a filler-free natural rubber composition with a cross-linking system. The remaining blends additionally contained hybrid fillers in amounts of 10, 20 and 30 phr. Hybrid fillers were mixtures of cereal straw with functional nano-additives, i.e., carbon black, silica or halloysite, in which the weight ratios were, respectively, straw:nano-additive 2:1 and 5:1.

### 2.2. Methods

**Figure 1:**
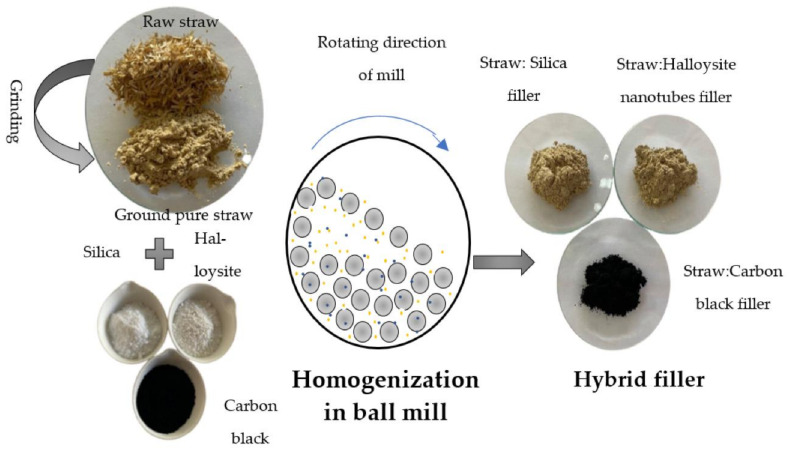
Scheme of straw hybridization with nano-additives.

Previously, the straw was ground with a Pulverisette 5 Classic Line planetary ball mill (Fritsch, Idar-Oberstein, Germany) (parameters of grinding: time 2 h, speed 300 rpm) and dried at 70 °C until a constant mass was obtained (approx. 2 days). To obtain two-component fillers, straw with each individual nano-additive was hybridized. Mechanical modification of the biofiller was carried out with the use of a Pulverisette 5 planetary mill (Fritsch GmbH, Idar-Oberstein, Germany). Hybridization was performed for 15 min at 300 rpm. Each of the straw:nano-additive systems were prepared in two weight ratios: 5:1 and 2:1 (Figure 1). The composition of the fillers is presented in Table 2.

Thermal analysis of hybrid fillers was carried out in a range of temperature of 25–700 °C. Up to 600 °C, measurements were made in argon atmosphere (55 mL/min), while from 600 °C to 700 °C, the pyrolysis residue was burnt in air (55 mL/min). The heating rate of both steps was 20 °C/min. Measurements were recorded using a TGA analyzer (Mettler Toledo, Greifensee, Switzerland).

The oil absorption parameter (DBPA) was determined using a Brabender Absorptometer C (Brabender, Duisburg, Germany). The test was based on the measurement of the torque while mixing the filler (20 g) with simultaneous dosing of n-dibutyl phthalate (DBP). Titrant was dosed at a constant rate of 4 mL/min. The result (DBPA) is the amount of absorbed titrant (per 100 g of filler) corresponding to the maximum torque. The test was performed according to the ASTM D2414 standard.

Measurement of the contact angle of the straw materials with water was carried out on pressed filler discs. An OCA 15EC goniometer (DataPhysics Instruments GmbH, Filderstadt, Germany) was used to place a drop of distilled water (10 µL) on the smooth surface of the pressed fillers. Then, just after the water drop was deposited, a picture was taken. Based on the drop shape analysis using the software, the value of the contact angle was determined.

Samples of hybrid fillers were analyzed using an LEO 1530 Gemini scanning electron microscope (Zeiss, Oberkochen, Germany).

The process of making rubber mixtures was in two stages. In the first one, NR was plasticized (4 min) and mixed with the filler (4 min). Mixing was carried out using a laboratory blender (Brabender, Duisburg, Germany) at 50 °C with a rotational speed of 40 rpm. In the second stage, the system of vulcanizing components was mixed in using laboratory rolling mills.

In order to determine the optimal cross-linking time (t_90_) and rheometric parameters of elastomeric composites, vulcanization curves were recorded using an Alpha Technologies rheometer (Alpha Technologies, Hudson, OH, USA).

Samples of vulcanizates were prepared by pressing elastomer mixtures with the use of hydraulic presses under a pressure of 15 MPa at 160 °C, with time t_90_.

Based on the measurements of the equilibrium swelling of the samples in toluene, the cross-linking density of the vulcanizates was calculated. The calculations were made according to the Flory–Rehner equation [56] (Equation (1)).

(1) $$\gamma_{e}=\frac{\ln\left(1-V_{r}\right)+V_{r}+{\mu V}_{r}^{2}}{V_{0}(V_{r}^{\frac{1}{3}}-\frac{V_{r}}{2})}$$

$\gamma_{e}$—the cross-linking density (mol/cm^3^), V_0_—the molar volume of solvent (toluene: 106.7 cm^3^/mol). µ—the Huggins parameter of the polymer–solvent interaction—was calculated from Equation (2) [57]:

(2) $$\mu =\mu_{0}+\beta \cdot V_{r}$$

µ_0_—the parameter determinant of non-crosslinked polymer/solvent relations, β—the parameter determinant of cross-linked polymer/solvent relations (µ_0_ = 0.478, β = 0.228), Vr—the volume fraction of elastomer in the swollen gel (Equation (3)) [58].

(3) $$V_{r}=\frac{1}{1+Q_{w}\frac{\rho_{r}}{\rho_{s}}}$$

ρ_r_—natural rubber density (0.99 g/cm^3^), ρ_s_—solvent density (toluene: 0.86 g/cm^3^), Qw—the weight of equilibrium swelling:

(4) $$Q_{w}=\left(\frac{m_{\mathrm{sw}\;}-m_{s}}{m_{s}}\right)\cdot \left(\frac{100+x}{100}\right)$$

m_sw_—the weight of the swollen sample; m_s_—the weight of the dry sample; 100—the elastomer content in the sample; x—the filler content in the sample.

Mechanical properties of vulcanizates, including stress at 100, 200 and 300% elongation (Se_100,200,300_), tensile strength (TS) and elongation at break (Eb), were determined using a universal tensile strength tester (ZwickRoell, Ulm, Germany). The testing machine was equipped with an extensometer measuring elongation during deformation. Dumbbell-shaped samples with a width (on the measuring section) of 4 mm and a thickness of 1 mm were used for the tensile strength test. The crosshead speed was 500 mm/min. The measurement was performed in accordance with ISO 37 [59].

The digital microcomputer-controlled hardness tester Shore A type (ZwickRoell, Ulm, Germany) was applied for determining the hardness of rubber vulcanizates to ISO 48 [60] Hardness values were calculated after 10 measurements on each side of the sample.

The Payne effect (ΔG’) was determined on the basis of the decrease in the storage modulus during dynamic mechanical analysis. A rotational rheometer ARES-G2 (TA Instruments, New Castle, DE, USA) was used to carry out measurements. The dynamic rheological measurements were at a frequency of 10 rad/s by varying strain amplitude from 0.1 to 100%. The ΔG’ value was determined from Equation (5).

(5) $$\Delta G^{\prime }={G^{\prime }}_{\min}\left(\lim10^{-1}\right)-{G^{\prime }}_{\max}\left(\infty \right)$$

G’_min_(lim 10^−1^)—a composite storage modulus determined under a deformation of 0.1%;

G’_max_(∞)—a composite storage modulus determined under the max deformation.

The flammability of natural rubber composites was tested using a cone calorimeter (Fire Testing Technology Ltd., East Grinstead, UK). Vulcanizate samples with dimensions of 100 mm × 100 mm × 2 mm were tested in a horizontal position using a 35 kW/m^2^ incident heat flux.

The analyzer Jupiter STA 449F3 (Netzsch Company, Selb, Germany) was used to determine the thermal properties of elastomer composites. The measurement was carried out in air atmosphere, in a temperature range of 25–700 °C at a heating rate of 10 °C min^−1^.

## 3. Results and Discussion

### 3.1. The Oil Absorption Parameter (DBPA) Measurements of Filler

**Figure 2:**
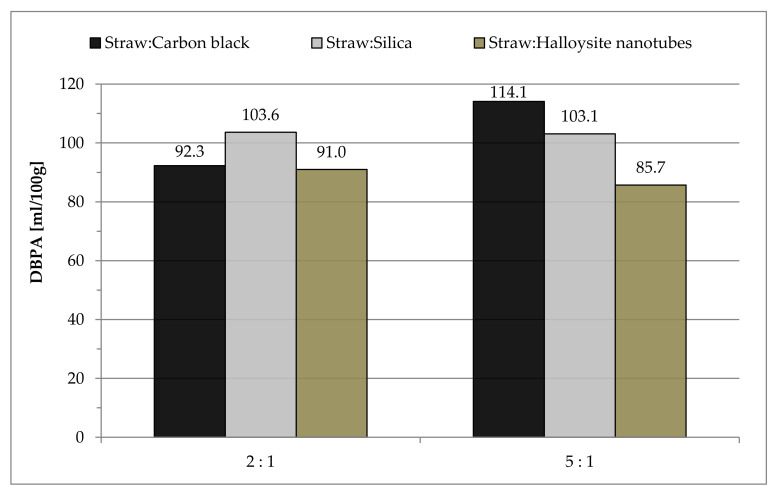
The DBPA (n-dibutyl phthalate absorption) value of hybrid filler.

The study of the absorption of n-dibutyl phthalate by hybrid fillers consisting of mixtures of straw with carbon black, silica and halloysite was carried out. The measurements aimed to determine the DBPA parameter, which characterizes the porosity of fillers and the ability of the fillers to form the so-called “own structure”. Figure 2 shows the DBPA parameter values for the prepared fillers.

The highest recorded value of n-dibutyl phthalate absorption was observed for the mixture of straw and carbon black in a weight ratio of 5:1, which may indicate the highest porosity and a greater dimension of voids between the formed aggregates of particles. The 2:1 straw to carbon black filler mixture was 19% lower. The more complex the structure, the greater the tensile strength, lower the elongation at break and the better the tear resistance of vulcanizates. On the other hand, the increased agglomeration of fillers in the elastomer matrix, resulting from the weak interaction between the rubber and the filler, causes the deterioration of properties.

In the case of hybrid straw fillers with silica, no significant change in the absorption of n-dibutyl phthalate was shown depending on the weight ratio used. The DBPA value was on average 10% lower than the highest recorded during the test (sample Straw:Carbon black_5:1).

The lowest amounts of absorbed dibutyl phthalate were recorded for mixtures of straw with halloysite nanotubes. The DBPA value was the lowest for the sample with the lower content of halloysite nanotubes, amounting to 85.7 mL/100 g (Figure 2).

In this case, the influence of the architecture of filler particles on the tested properties of biocomposites should be taken into account, as carbon black and silica particles have a spherical shape, and halloysite has a tubular structure. Moreover, the specific surface area and aspect ratio also affect the varying degree of “packing” of the particles.

### 3.2. The Contact Angle Measurements of Straw-Based Fillers

**Figure 3:**
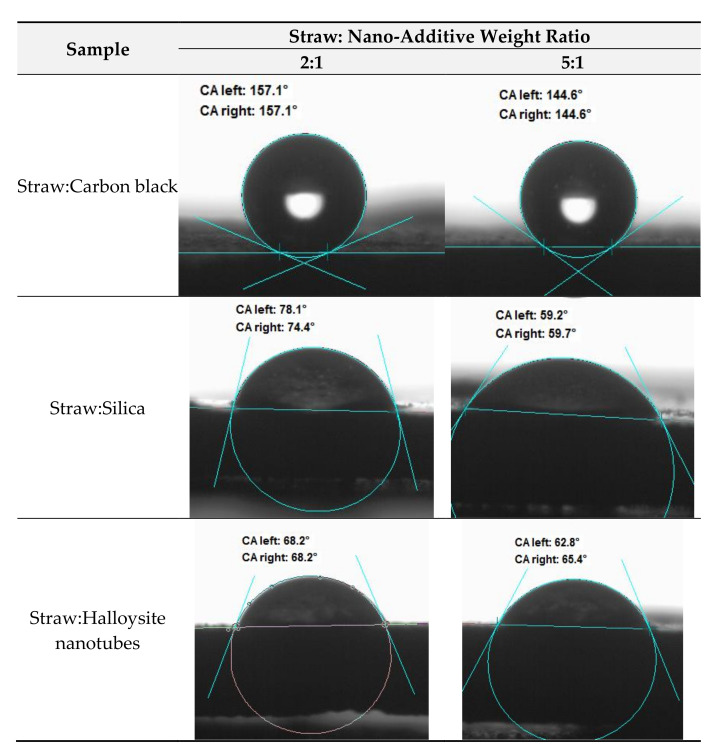
The contact angle (CA) measurements of hybrid fillers.

Measuring the wettability of powders is a difficult task due to liquid absorption during the experiment. Nevertheless, the method of measuring the contact angle (CA) on a compressed powder is widely used and acceptable [61]. Contact angles on “non-ideal” surfaces, such as compacted powders, depend on interfacial tensions but also on many other factors such as surface roughness, chemical heterogeneity, adsorbed layers, molecular orientation, swelling and the partial dissolution of components in material. As far as the surface properties of the obtained powders are concerned, the measurements carried out are indicative (Figure 3). The straw fillers with the addition of carbon black (contact angle of water on compressed filler disks was 157°–144°) were characterized by the highest hydrophobicity. Powders containing straw hybridized with silica (CA = 78°–59°) and halloysite (CA = 68°–62°) showed much more hydrophilic properties. The silanol groups present on the silica surface have a positive effect on their separation with polymers. However, their presence causes an increase in the hydrophilicity of the surface due to its wetting and, consequently, water sorption [62]. Unlike other nanoclays, the multilayer tubular structure of halloysite nanotubes (HNTs) is related to their relatively weak hydrophilic nature. Most of the hydroxyl groups (Al-OH) are inside the pipes, while only a few hydroxyl groups (Al-OH and Si-OH) were found at the edges of the outer surfaces of the pipes [63]. Therefore, the addition of halloysite nanotubes should have a positive effect on its dispersion in non-polar natural rubber. In all investigated cases, the increase in straw content in the hybrid fillers contributed to the decrease in the water contact angle of the fillers’ surface. Straw as a material of lignocellulose can annotate water and thus has a strongly hydrophilic character.

### 3.3. Surface Morphology of Hybrid Biofillers

**Figure 4:**
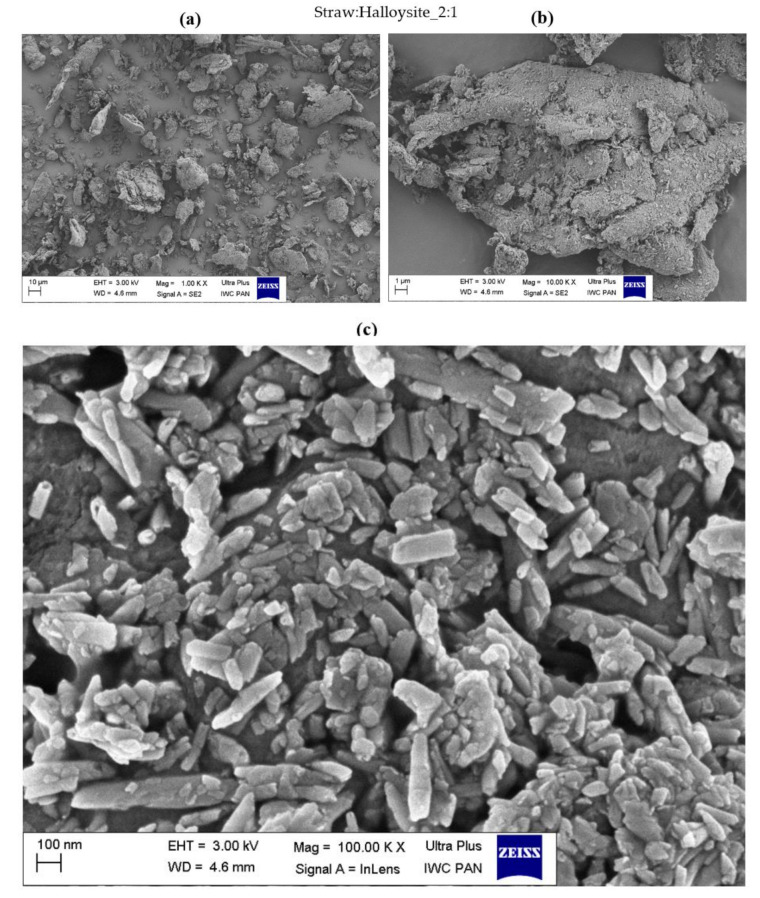
Microscopic images of straw fillers with the addition of halloysite nanotubes (2:1) at a magnification of (**a**) ×1.00 k, (**b**) ×10.000 k, (**c**) ×100.00 k (EHT is electron high tension; WD is working distance).

**Figure 5:**
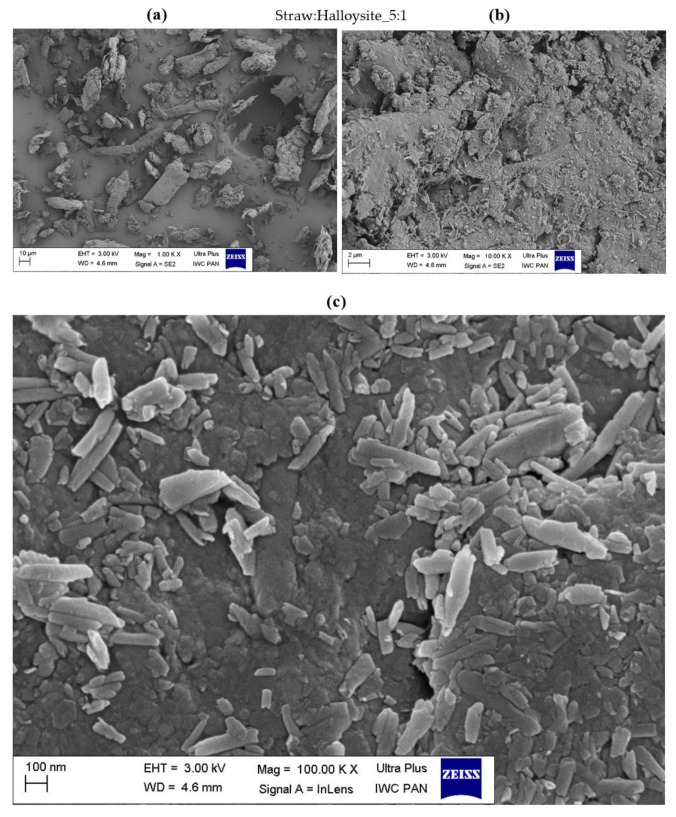
Microscopic images of straw fillers with the addition of halloysite nanotubes (5:1) at a magnification of (**a**) ×1.00 k, (**b**) ×10.000 k, (**c**) ×100.00 k.

**Figure 6:**
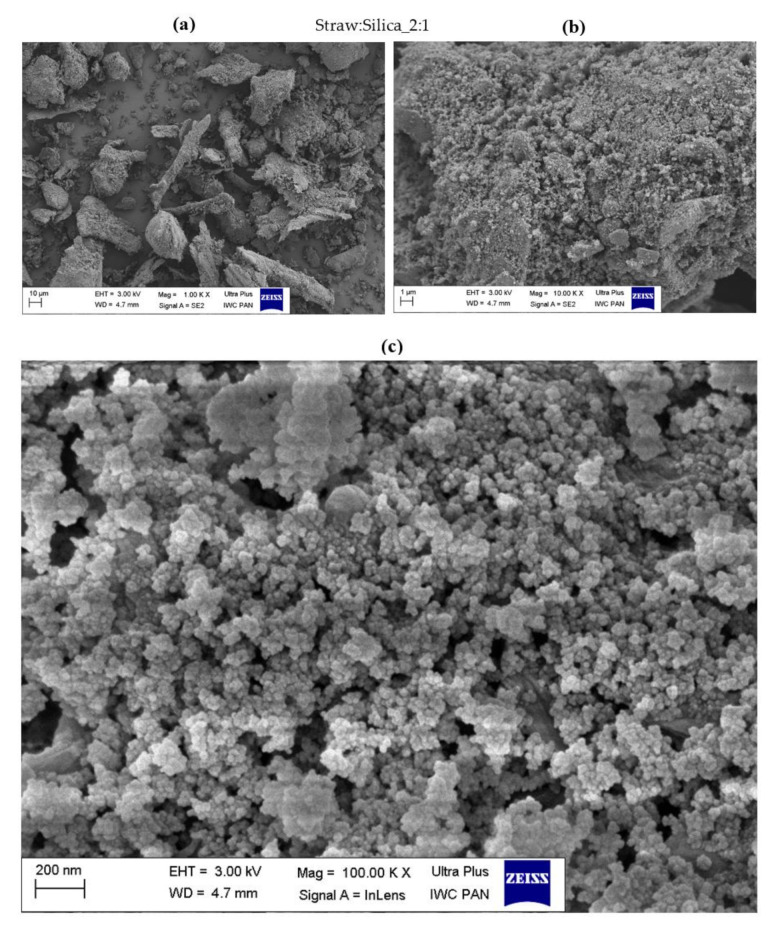
Microscopic images of straw fillers with the addition of silica (2:1) at a magnification of (**a**) ×1.00 k, (**b**) ×10.000 k, (**c**) ×100.00 k.

**Figure 7:**
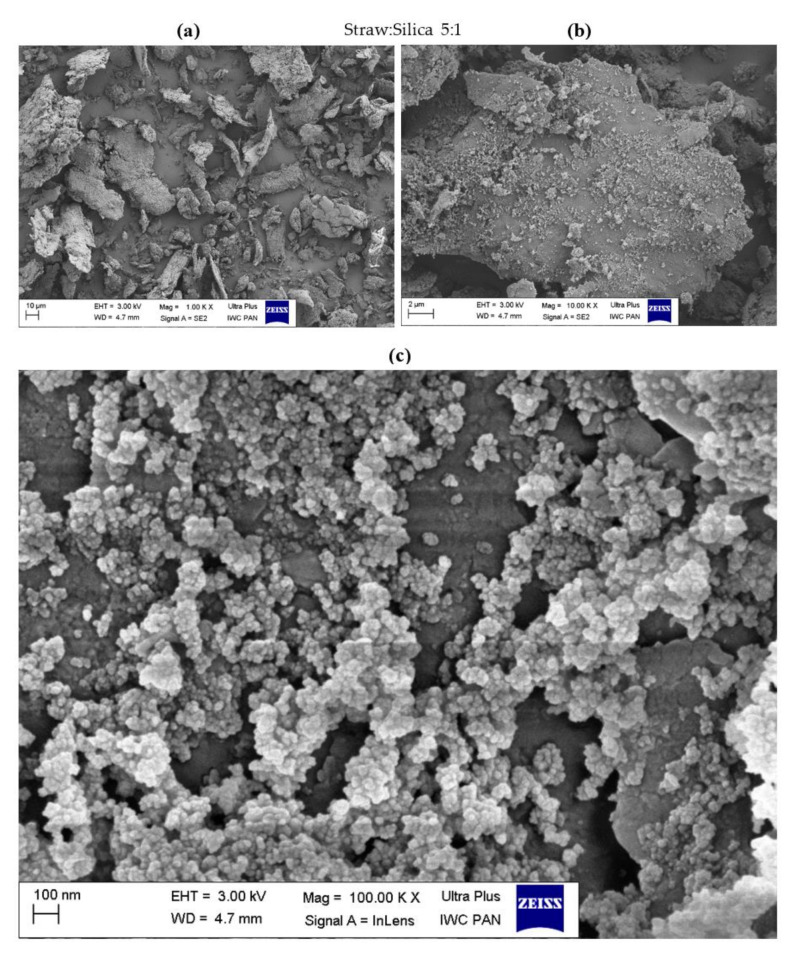
Microscopic images of straw fillers with the addition of silica (5:1) at a magnification of (**a**) ×1.00 k, (**b**) ×10.000 k, (**c**) ×100.00 k.

**Figure 8:**
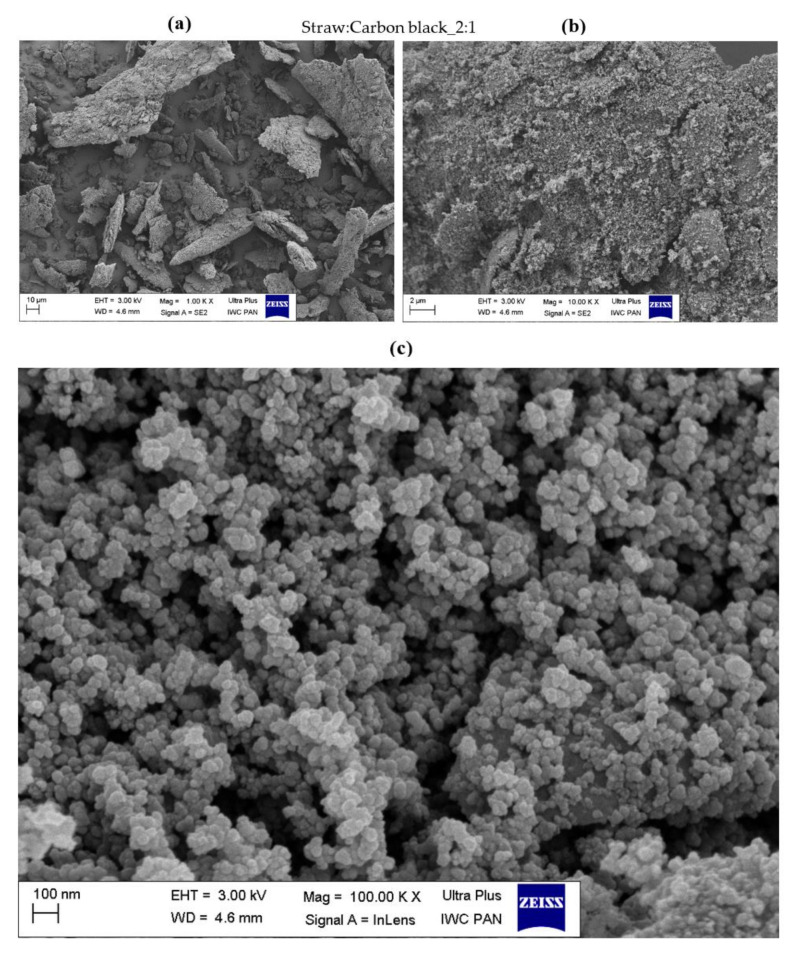
Microscopic images of straw fillers with the addition of carbon black (2:1) at a magnification of (**a**) ×1.00 k, (**b**) ×10.000 k, (**c**) ×100.00 k.

**Figure 9:**
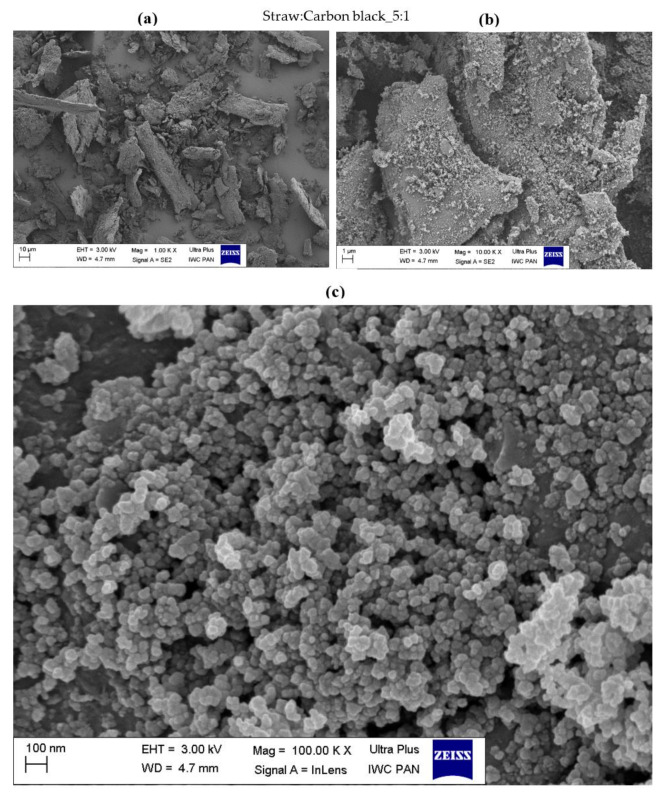
Microscopic images of straw fillers with the addition of carbon black (5:1) at a magnification of (**a**) ×1.00 k, (**b**) ×10.000 k, (**c**) ×100.00 k.

The morphology of the obtained straw fillers was assessed based on the SEM images presented in Figure 4, Figure 5, Figure 6, Figure 7, Figure 8 and Figure 9. When analyzing the obtained images, it can be seen that in all cases, the straw was present in the form of particles of different sizes and shapes. It had the form of thin plates ranging in size from several dozen to several micrometers. The variety of the obtained straw particles after grinding, i.e., mechanical modification, could be explained based on their structure. The larger straw particles could be unbroken elements of the cell wall. The wheat straw consisted mainly of cell walls in which cellulose was one of the main components. In the cell walls, parallel chains of cellulose were hydrogen bonded to form microfibrils, which were the basic unit of the plant cell wall structure and were characterized by high strength [64]. They greatly contributed to the mechanical strength of the plant cell walls and acted as their skeleton, which greatly hindered their breakdown. The microfibrils were linked by a gel matrix composed of hemicelluloses, lignin and other carbohydrate polymers. They were much less durable, which in turn facilitated the fragmentation of the material. In the case of nano-additives, it was observed that their particles were homogeneously deposited on the straw surface. Besides, the images with the largest magnification (Figure 4, Figure 5, Figure 6, Figure 7, Figure 8 and Figure 9c) showed the architecture of the nano-additives used well. Thus, they confirm that the structure was not damaged during mechanical modification. Halloysite particles were in the form of oblong tubes, while silica and carbon black were in the form of spherical particles.

Generally, in the case of hybrid fillers containing halloysite (Figure 4c and Figure 5c), nanometric halloysite structures evenly distributed over the straw surface were visible. In the case of carbon black and silica, the particles showed a greater tendency to aggregate to form agglomerates. Nevertheless, single (primary) particles were also visible on the surface of the biofiller (Figure 6c, Figure 7c, Figure 8c and Figure 9c).

### 3.4. Thermal Properties of the Materials

**Figure 10:**
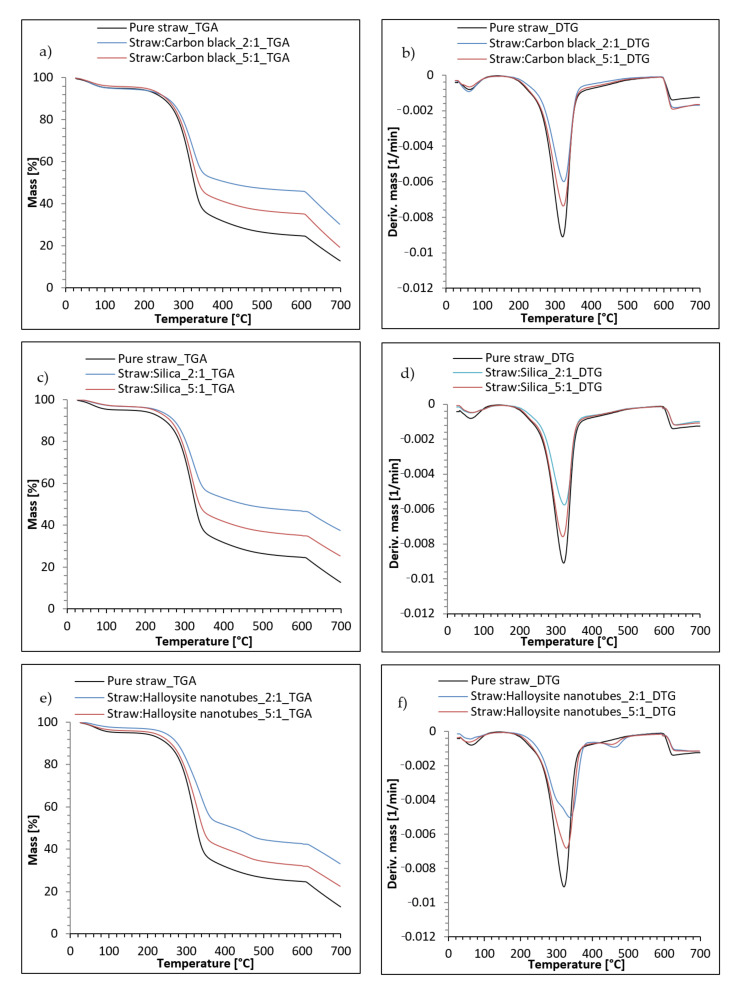
Thermal analysis of biofillers: (**a**) TG curve and (**b**) DTG curve for straw:carbon black; (**c**) TG curve and (**d**) DTG curve for straw:silica, (**e**) TG curve and (**f**) DTG curve for straw:halloysite nanotubes.

Figure 10 shows the course of the TG (thermogravimetry) and DTG (derivative thermogravimetry) curves of the fillers based on straw. During the decomposition of the materials on the DTG curves, peaks related to the ranges in which there was an intense mass loss were observed. In each case, the fillers were decomposed in several stages. For all the fillers, a peak was recorded in the temperature range of 50–120 °C. The loss of mass in this region corresponded to the evaporation of the moisture contained in the samples. The endothermic peak corresponded to the loss of physically adsorbed water and interlayer water. Pure_Straw had the greatest ability to absorb water, which was confirmed by thermal analysis by recording the greatest loss of mass in the range of these temperatures. In the next stage, covering a wide temperature range, the filler decomposed mainly due to the degradation of the lignocellulosic material. At this stage, in the temperature range of 180–500 °C, thermal degradation of the main straw composition (cellulose, hemicellulose and lignin) began [65]. In the case of fillers containing halloysite, a slight endothermic peak was observed in the region of 430–500 °C, related to dehydroxylation [66]. In this region, the release of OH groups from the octahedral coordinated Al^3+^ ion and the formation of a “metalloysite” took place [67]. In the case of carbon black and silica, which were components of the remaining fillers, no changes were noted in the tested range. This was because they were stable at these temperatures.

Table 3 presents the numerical data concerning the thermal analysis of the tested fillers. The temperature at which a 5% weight loss was recorded was assumed as the beginning of the thermal decomposition of straw. The hybrid fillers had a much higher T_5_ temperature value when entrained from pure straw, for which the value was 146 °C. Straw:nano-additive 5:1 materials showed values of the initial decomposition temperature oscillating around 200–222 °C. On the other hand, for the samples of straw:nano-additive in a weight ratio of 2:1, the T_5_ parameters were higher by 84, 59 and 96 °C, respectively, for silica, carbon black and halloysite compared to pure straw. In the case of the temperature at which 50% weight loss was registered, the same relationship was observed as for the T_5_ parameter. The T_50_ value for pure straw was the lowest at 327 °C. For hybrid fillers (straw:nano-additive 5:1) the values were slightly higher and were between 338 and 349 °C. On the other hand, for straw:nano-additive 2:1 materials, higher temperatures of 50% weight loss (by about 100 °C) were observed compared to the pure straw sample. Summing up, the thermal stability of two-component fillers was significantly improved by the hybridization of straw with the addition of silica, carbon black and halloysite, which were thermally resistant and did not decompose in the discussed temperature range.

After the pyrolysis process from 600 °C to 700 °C, the sample was burnt in an oxygen atmosphere. The solid residue after combustion (R_700_) for pure straw was 13%. This residue consists mainly of inorganic compounds present in straw, such as compounds of silicon, calcium, magnesium, etc.

In the case of hybrid fillers, the residue after combustion was much higher, because the samples contained a thermally stable nano-additive that did not degrade in the tested temperature range. The highest R_700_ value was found for hybrid straw fillers containing silica. A lower value of the residue was observed for the straw:carbon black fillers, which resulted from the fact that carbon black contained a large amount of bound water, which evaporated in the initial stage of pyrolysis. Hybrid fillers with halloysite were characterized by the lowest amount of solid residue. In this case, the processes of dehydration and dehydroxylation of HNT took place during pyrolysis, which was associated with the separation of water from the sample, which resulted in an additional loss of filler mass.

### 3.5. Rheometric Properties and Optimal Curing Time of Rubber Mixtures

**Figure 11:**
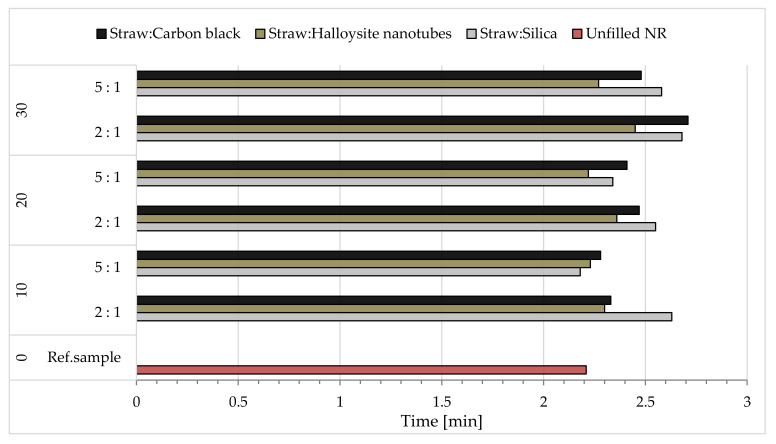
The optimal curing time of rubber mixtures.

The influence of the type and content of the hybrid fillers used on the rheometric parameters of the obtained elastomer blends was investigated. On their basis, a comparison was made with the reference mixture, which is unfilled natural rubber. The results of the minimum (M_min_) and maximum (M_max_) torque and its increase ΔM during vulcanization are presented in Table 4, while the determined optimal cross-linking times (t_90_) are presented in Figure 11.

Based on the above results, it was possible to conclude that, regardless of the type of filler introduced into the elastomer matrix, the maximum torque M_max_ increased relative to the reference blend, which was directly related to the increase in the stiffness of the composition. Moreover, increasing the content of hybrid fillers led to higher values of this parameter.

The change in the increase in torque ΔM during vulcanization proceeded with the same dependence: increasing the content of fillers in the composite results in higher values of this parameter (Table 4). Considering the effect of the type of filler used, greater increases in torque could be observed in the case of composites containing carbon black and halloysite, which could indicate a more developed structure of these fillers in the composites. Smaller, although still higher than for the reference mixture, ΔM values were recorded for the mixtures containing silica. The increase in torque is an indirect measure of the cross-linking density, so probably the efficiency of the vulcanization process in these compositions was lower and was related to the phenomenon of absorption of the components of the cross-linking complex (e.g., vulcanization activators or accelerators) by the silica [68].

When analyzing the results of the minimum torque, which is a measure of the viscosity of the composition, higher M_min_ values were observed, especially with a higher silica content in the system. This was probably due to insufficient dispersion of the additive in the elastomer matrix.

Optimal cross-linking times of the tested compositions were greater than or equal to the corresponding reference mixture (Figure 11). Only for the sample containing 10 phr of the hybrid filler consisting of straw and silica with a weight ratio of 5:1 was the time slightly shorter. Moreover, it was observed that the greater the proportion of fillers in the compositions, the longer the vulcanization process, which was a consequence of the agglomeration of the fillers in the elastomer matrix. On the other hand, when considering the type of hybrid filler used, it could be noticed that the mixtures containing halloysite had shorter cross-linking times.

### 3.6. The Cross-Linking Density of Natural Rubber Vulcanizates

**Figure 12:**
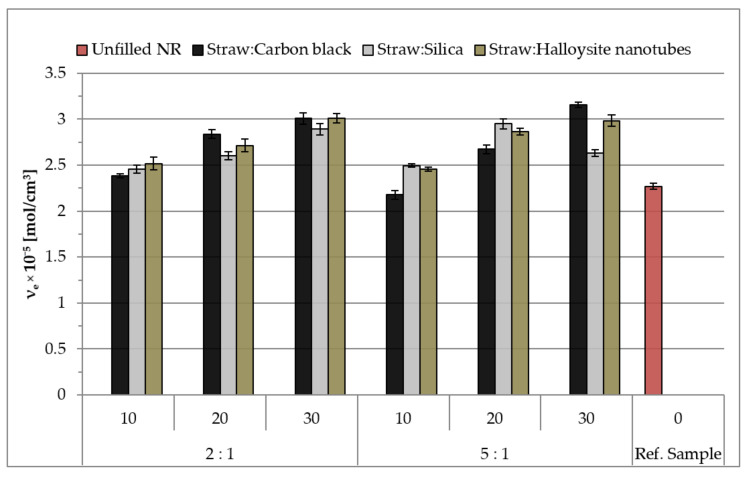
The density of network nodes of vulcanizates.

The effect of the type and content of the hybrid fillers used on the efficiency of the cross-linking process was determined by the density of network nodes using the equilibrium swelling method (υ_e_). The results presented in Figure 12 show that regardless of the type of the introduced filler, the degree of cross-linking was higher compared to the reference sample. The reason for the increase in the analyzed parameter was the fact that, in the case of biocomposites, apart from the chemical cross-links of the rubber with the cross-linking substance, there were also chemical and physical interactions of the polymer matrix with functional groups on the fillers’ surface. The result of these interactions were subsequent nodes of the spatial network.

Vulcanizates containing straw hybridized with carbon black and halloysite were characterized by a higher degree of cross-linking than the samples with the addition of silica. These observations correlate with the results of rheometric properties, where the highest values of the increase in torque were also shown by carbon black and halloysite samples. Increasing the number of physical network nodes increased the stiffness of the composition and thus generated a greater increase in ΔM during cross-linking.

It was also observed that, in contrast to vulcanizates containing carbon black, where relatively high values of υ_e_ were achieved with a higher degree of filling (20 and 30 phr), halloysite resulted in obtaining a high density of network nodes even at low filling (10 phr). For samples containing straw with added silica, the cross-linking density initially increased and then decreased sharply at 30 phr (Straw:Silica_5:1) (Figure 12). This effect could be related to the agglomeration of the filler in the elastomer matrix or its absorption of the components of the cross-linker, which consequently led to a reduction in the efficiency of the vulcanization process.

### 3.7. The Payne Effect of Biocomposites

**Figure 13:**
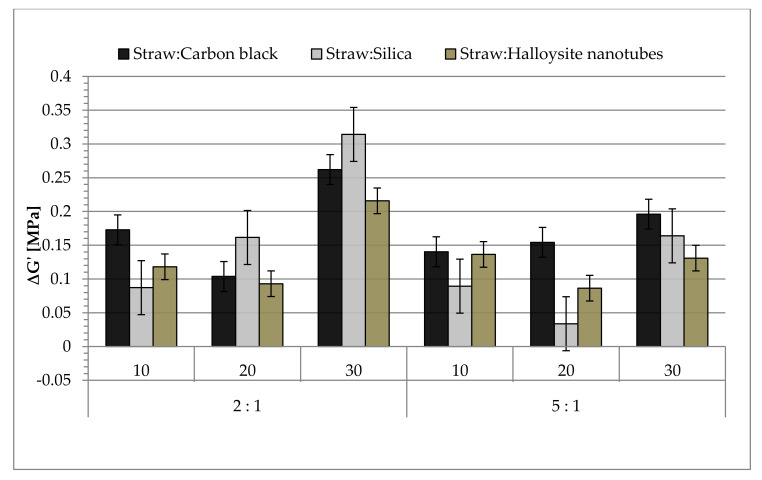
The Payne effect values of filled natural rubber.

In the prepared samples of biocomposites, the Payne effect values were determined as a change in the value of the storage module under conditions of the increasing amplitude of shear strains. The results of the calculations are presented in Figure 13. The Payne effect occurred in the case of filled rubber mixtures, in which the formed secondary filler structures were destroyed. It was closely related to the interactions between the filler particles (filler–filler) and the filler and elastomer (filler–polymer) particles [69]. On the other hand, the ΔG‘ values also increased in the case of the formation of excessively large agglomerates of filler particles, which were the places of stress concentration.

Analyzing the results, it can be concluded that the use of filling at the level of 30 phr caused a significant agglomeration of all tested hybrids, hence high values of the Payne effect were observed. These observations correlated with the results of the tensile strength measurements, where the deterioration of the mechanical strength was also noted when the rubber mixtures were highly filled (30 phr), probably due to premature rupture of the sample as a result of stress concentration in the agglomerates. Silica vulcanizates showed the greatest tendency to agglomerate in the elastomer matrix, and the smallest samples were prepared with the use of halloysite. The obtained results confirmed the previously discussed results of the rheometric properties and determination of the concentration of nodes in the biocomposite network.

Taking into account the results of tensile strength measurements and dynamic mechanical analysis, it can be concluded that the most favorable reinforcing effect resulting from the extensive secondary structure of fillers, in which the agglomerates are not large enough to propagate an unfavorable stress concentration and, consequently, deterioration of the composite strength, was obtained at 10–20 phr of filler in the composition (Figure 13).

### 3.8. Mechanical Properties of Vulcanizates Containing Hybrid Fillers

The main factor determining the mechanical properties of rubber composites is the mechanism of stress transfer by the filler particles dispersed in the elastomer matrix. Thus, surface functional groups that determine the strength of the interaction between the components, the shape of the particles and the amount of added filler and other properties, e.g., the ability to create its own structure, i.e., a three-dimensional lattice, are important.

**Figure 14:**
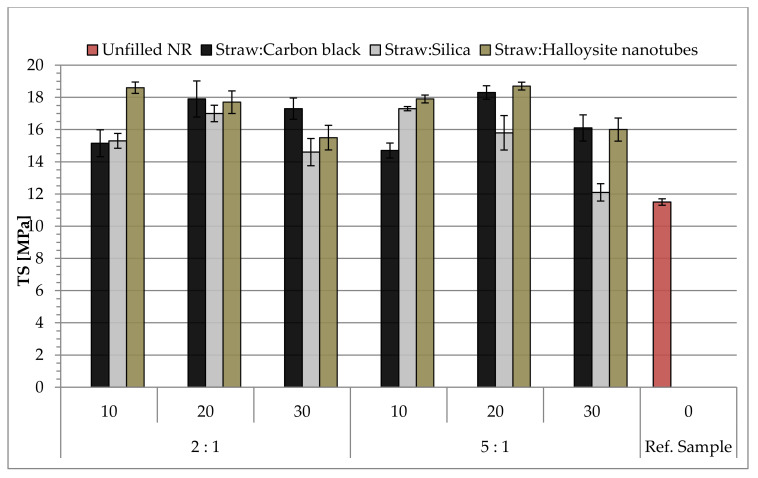
Tensile strength (TS) values of composites filled with straw:nano-additive materials.

**Figure 15:**
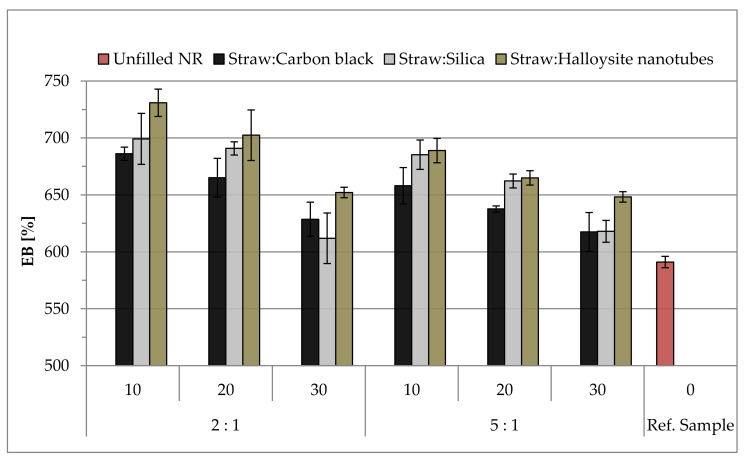
The elongation at break (Eb) parameters of natural rubber vulcanizates.

The influence of the type and content of hybrid fillers on the mechanical properties under static conditions was assessed based on the results of the measurements of the tensile strength (TS), relative elongation at break (Eb) and stress at 100, 200 and 300% elongation (SE_100_, SE_200_ and SE_300_). The recorded results are presented in Table 5 and graphs (Figure 14 and Figure 15).

The addition of fillers increased the relative modules SE_100_, SE_200_ and SE_300_. Moreover, the higher the content of fillers in the compositions, the higher the values of the parameters considered. The highest and more than twice higher values of the relative modulus in relation to the reference sample were observed in rubber mixtures containing carbon black (Straw:Carbon black_5:1_30phr). The samples with the addition of carbon black in the case of both considered weight ratios of fillers and their content in the mixtures showed higher values compared to the compositions containing silica or halloysite. As a consequence, the straw:carbon black hybrid filler was characterized by the greatest reinforcing effect (in the case of low deformation), which correlated with the obtained results of the DPBA (n-dibutyl phthalate absorption) analysis.

Analyzing the data presented in Figure 14, illustrating the results of the tensile strength (TS) measurements, it was observed that the use of a mixture of straw and halloysite, in contrast to the other tested hybrid fillers, causes the greatest strengthening effect even at low contents (10 and 20 phr in the rubber mixture). The optimal amount of the mixture of straw and carbon black in the analyzed composition was 20 phr, regardless of the weight ratio of both components of the hybrid filler. The probable cause of the poorer strength when using more hybrid fillers is the formation of agglomerates of their particles, which are the places of stress concentration in the composite and they consequently cause worse dispersion of the reinforcement in the elastomer matrix. The silica-containing vulcanizates generally showed lower tensile strength than the other materials (excluding the reference sample for which the lowest TS value was recorded), despite the strong ability to form its own structure in the elastomer matrix. This is because silica particles show a stronger tendency for interactions of the filler–filler type, which in turn, with its higher content, led to the increased agglomeration of this filler.

The use of hybrid fillers increased the relative elongation at break (Eb). The higher straw content in the compositions resulted in less flexibility of the samples (5:1 weight ratio). Regardless of the type of the analyzed hybrid filler, along with the increase in its content in the composition, the stiffness of vulcanizates increased, which in turn resulted in a reduction of Eb. Halloysite vulcanizates showed the greatest flexibility (Eb is even over 700%). This may be because, during stretching, the oblong particles of the halloysite oriented towards the acting force, which favored a greater elongation of the samples (Figure 15).

### 3.9. Hardness of Biocomposites

**Figure 16:**
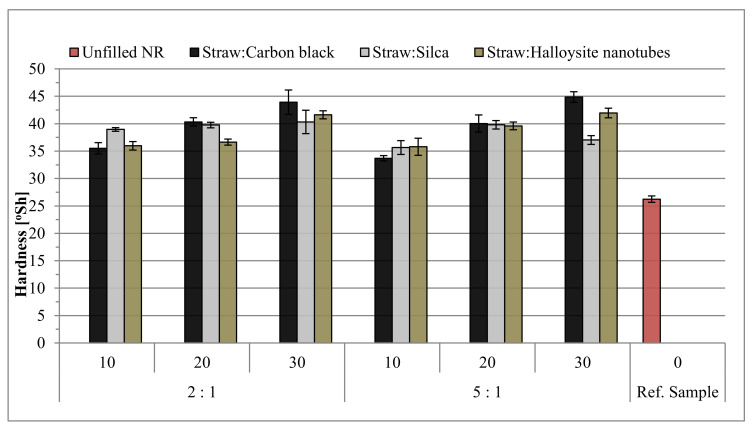
The hardness results of rubber vulcanizates.

The determination of the mechanical properties also included the measurement of the hardness of vulcanizates, and the results are presented in Figure 16. As expected, the addition of fillers increased the hardness by 30–70% compared to the reference mixture. Additionally, increasing the filling of the rubber mixtures determined a greater increase in hardness, which was also reflected in the results of cross-linking density, where the same relationship was also observed. The highest hardness was characteristic of vulcanizates with the addition of carbon black, for which it was possible to achieve as much as 55°Sh. Usually, the lowest hardness at a given filling level and weight ratio of components was recorded for samples with the addition of the mineral filler—halloysite.

### 3.10. Thermal Analysis of Natural Rubber Vulcanizates

**Figure 17:**
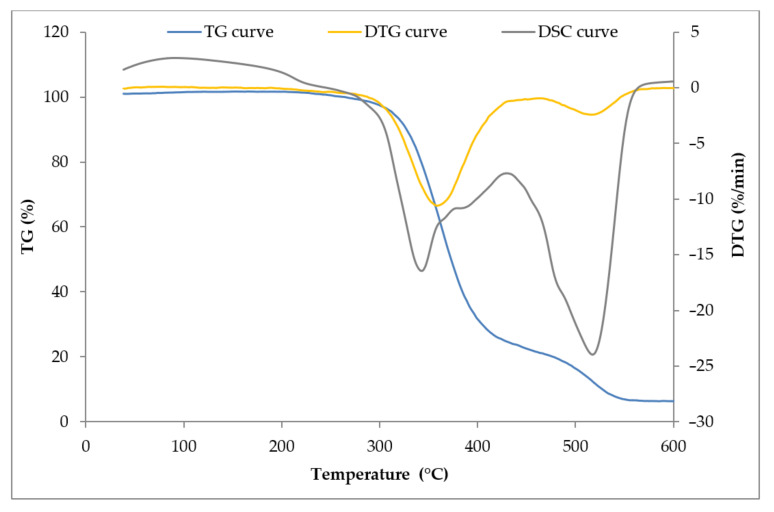
Thermal curves of unfilled vulcanizate (reference sample).

The thermal decomposition of cross-linked natural rubber took place in the temperature range ΔT = 310–465 °C, which was manifested in the exothermic peak recorded on the DSC (Differential Scanning Calorimetry) curve (Figure 17). This transformation was complex. At first, compounds with a high H/C ratio were formed, and then a slightly slower cyclized, charred residue. The rate of the formation of the volatile products of destruction was much higher than the rate of oxygen diffusion into the reaction zone, which meant that the destructive processes of the cross-linked natural rubber took place in the presence of oxygen deficiency. When the rate of oxygen diffusion and the rate of decomposition were similar, the combustion of the solid, charred residue began, which was manifested in the exothermic peak recorded on the DSC curve in the temperature range ΔT = 465–568 °C. The residue after thermal decomposition, amounting to 6.35% of the original sample mass, indicated the susceptibility of the cross-linked NR to the cyclization and carbonization processes.

The introduction of the straw:carbon black filler into the natural rubber matrix did not affect the value of the thermal stability parameters of the tested vulcanizates. It should be noted that in the presence of the straw:carbon black filler, the value of the T_5_ parameter decreased, but both the T_50_ and T_RMAX_ values increased significantly, which indicated a positive effect of the straw:carbon black filler on the thermal stability of composites containing it (Table 6). It should also be noted that the straw:carbon black filler, especially in the 5:1 weight ratio (Straw:Carbon black_5:1), limited the rate of thermal decomposition of the tested composites (dm/dt parameter). This is due to the polymer–filler interaction mechanism, the consequence of which is the reduced segmental mobility of the elastomeric chains around the filler particles, as well as the fact that carbon black is a good radical scavenger. Its presence in the matrix of the composite inhibits free radical reactions while increasing the probability of the recombination of primary macro-radicals by extending their residence time in the cage, which positively affects the reduction of degradation processes and thermal destruction [49].

**Figure 18:**
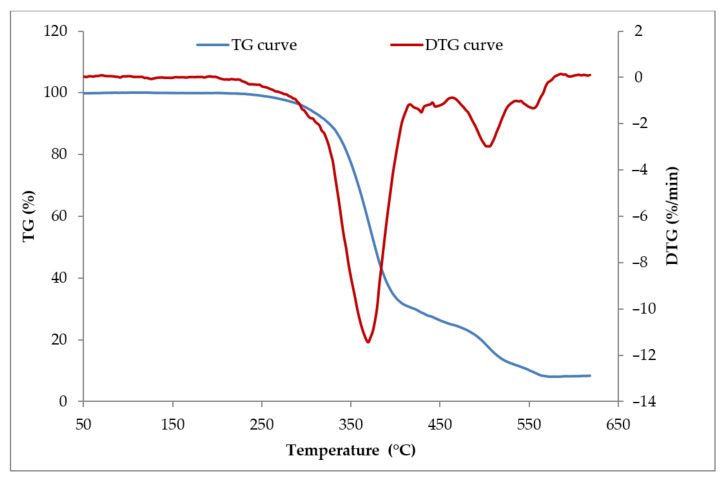
The TG and DTG curves of vulcanizates filled with 30 phr of Straw:Carbon black_2:1.

On the TG and DTG curves of composites containing the straw:carbon black filler, a two-stage combustion process of the residues after thermal decomposition was recorded (Figure 18). This was because the carbon black burned at temperatures above 500 °C, i.e., outside the thermal decomposition area of most elastomers (Table 6).

**Figure 19:**
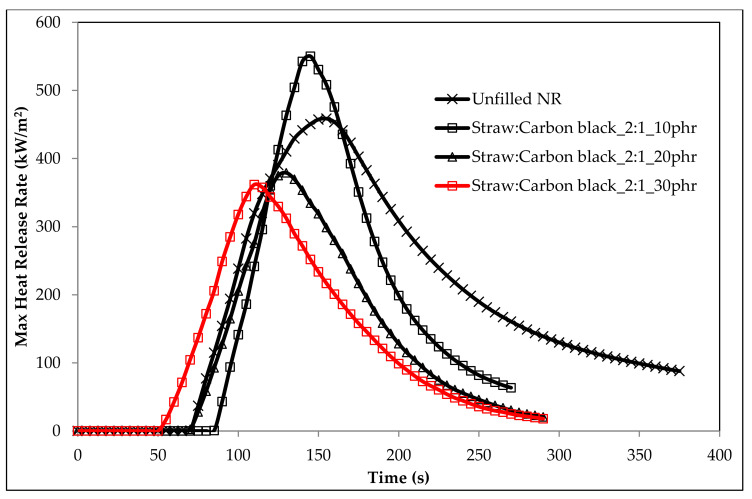
HRR_MAX_ curves of biocomposites containing Straw:Carbon black_2:1 fillers.

The introduction of a natural filler in the form of straw in combination with both halloysite and silica had a positive effect on the thermal stability parameters T_50_, T_RMAX_, dm/dt and P_650_. The literature review shows that aluminosilicates (e.g., halloysite) increase the amount of carbon layer formed. They act as insulators that hinder the diffusion of gaseous products of thermal decomposition into the flame, and at the same time constitute a barrier for oxygen diffusion into its interior (Table 6) [70,71,72]. It should be noted that the lowest dm/dt parameter values were recorded for the straw:halloysite nanotubes filler (Table 6). Undoubtedly, the barrier properties of halloysite played an important role in limiting the rate of thermal decomposition of composites containing the straw:halloysite nanotubes filler. Halloysite is impermeable to vapors and gases, therefore, under the combustion conditions of the composite, low-molecular-weight elastomer thermal decomposition products can diffuse outward only through strictly defined spaces between the dispersed halloysite tubes. Some of the liquid destructs are additionally immobilized inside the cylindrical structures of halloysite. As a result, the speed of the transport of volatile, including combustible, destructive products to the flame zone is significantly limited. It should be noted that the reduction of the dm/dt parameter took place both in the case of the Straw:Halloysite nanotubes_2:1 filler and the Straw:Halloysite nanotubes_5:1 filler, which indicated that the lignocellulosic filler had a significant effect on the reduction of the sample decomposition rate. Under the influence of straw:halloysite nanotubes, the share of cavitation and charring processes in the solid phase of sample combustion also increased (P_650_, Straw:Halloysite nanotubes_2:1). In the case of the straw:silica filler, its positive effect on the value of T_50_ and T_RMAX_ parameters was observed (Table 6). The flammability results obtained by the cone calorimetry method indicated that the introduction of the straw:carbon black filler into the matrix of cross-linked natural rubber limited its flammability, expressed by the parameters: heat release rate (HRR), maximum heat release rate (HRR_MAX_), time to maximum heat release rate (tHRR_MAX_), effective heat of combustion (EHC) or maximum average heat release rate (MARHE) (Figure 19, Table 7).

The straw:carbon black filler in the amount of 30 phr (Straw:Carbon black_2:1_30phr) reduced the HRR parameter value by 28.45% for the unfilled NR vulcanizate (unfilled NR), and the HRR_MAX_ parameter by 21.1%. The total heat release (THR) parameter was reduced by 46.3%, while the EHC and MARHE parameters by 50.2 and 27.4%, respectively (Table 7).

The limited flammability of the tested composites was due to several reasons. First of all, there are graphite layers on the carbon black surface. It is known that polymer chains are easily trapped by the edges of these structures. The increase in the immobilization of macromolecules on the surface of the carbon black filler reduces the amplitude of the thermal vibrations of the polymer and, consequently, inhibits its thermal decomposition. It should also be taken into account that there are paramagnetic centers on the surface of carbon black particles, which are stable radicals [49]. This confirms that in the processes of thermal degradation of composites, carbon black plays the aforementioned role of a free radical scavenger, which undoubtedly contributes to increasing thermal stability and reducing the flammability of the tested composites. It should also be taken into account that the carbon particles act as sorbents for volatile products of thermal decomposition of elastomers, and the larger the specific surface area of the carbon black, the greater its sorption capacity.

**Figure 20:**
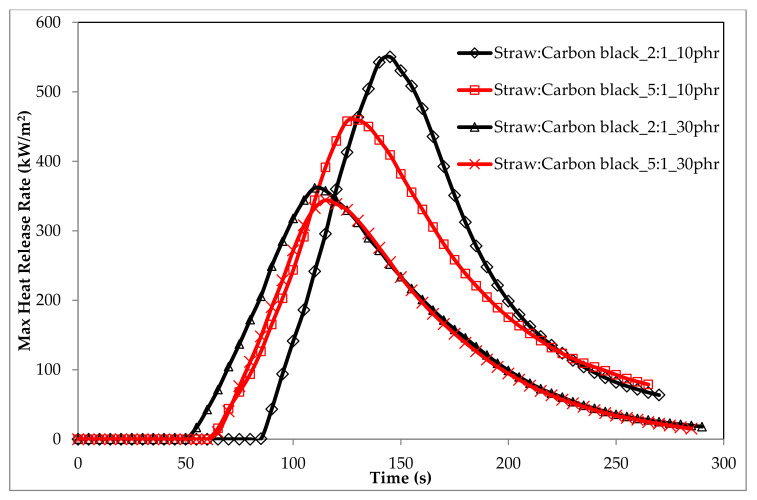
Comparison of HRR_MAX_ curves containing the straw:carbon black filler in a weight ratio of 2:1 and 5:1, respectively.

**Figure 21:**
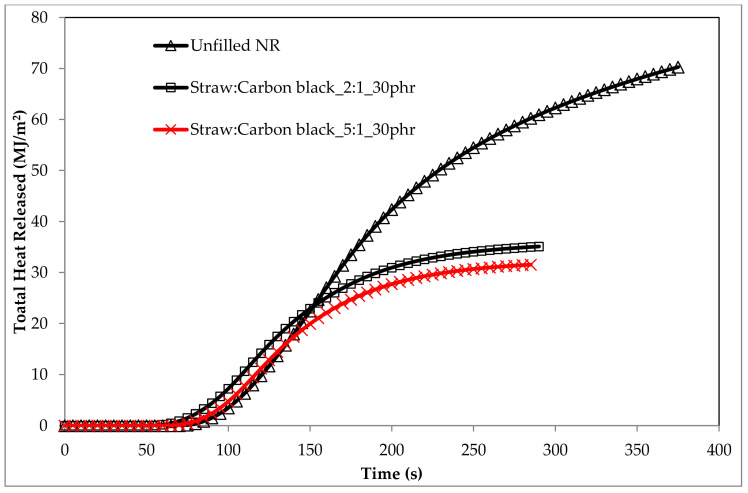
The THR curves of vulcanizates filled with 30 phr of straw:carbon black fillers.

The data in Table 7 show that the composites containing the Straw:Carbon black_5:1 were less flammable than the composites containing the Straw:Carbon black_2:1 filler (Figure 20 and Figure 21).

The flammability of the natural filler, i.e., lignocellulosic fibers, depends on their chemical composition and degree of polymerization and crystallization, as well as the orientation of microfibers, i.e., fibrils. The literature review shows that even a small amount of lignin (10%) increases the resistance of natural fibers to fire [73,74]. During the decomposition of lignin in the temperature range ΔT = 250–400 °C, phenolic rings were formed as a result of the dissociation of ether and carbon–carbon bonds, which were responsible for the increase in the amount of carbon residue with insulating properties. On the other hand, the influence of the high cellulose content on the flammability of the lignocellulosic filler was ambiguous. On the one hand, during the decomposition of cellulose-rich fibers (high degree of crystallinity of the fiber), a large amount of levoglucosan (liquid intermediate) was formed which may increase the flammability of the fiber. On the other hand, it should be noted that the high cellulose content in the natural fiber required a higher activation energy value for the destruction of the crystal structure of cellulose, which in turn increased the ignition temperature of the filler.

The orientation of the fibrils primarily controls the amount of oxygen that diffuses into the fiber. The greater the orientation of the fiber, the lower its gas permeability. Given the above, it should be stated that natural fibers with a low degree of crystallinity and a high degree of polymerization and fibril orientation are an excellent filler of polymeric materials from the point of view of reducing their flammability. The sample containing the straw:carbon black filler in the amount of 30 phr (Straw:Carbon black_5:1_30phr) was characterized by the lowest flammability of all tested samples containing straw:carbon black filler, which was confirmed by the high fire suppression potential of the natural filler used (Figure 20 and Figure 21, Table 6 and Table 7).

A significant degree of reduction in the flammability of the tested composites was also achieved with the use of the straw:halloysite nanotubes filler. The anti-pyrenic effect of halloysite, in addition to the abovementioned barrier effect, was associated with its endothermic dehydration and dehydroxylation processes occurring in the temperature range ΔT = 120–560 °C. The water released as a result of endothermic transformations of halloysite diluted the gaseous, combustible products of the composite destruction. Moreover, halloysite in the temperature range ΔT = 885–1000 °C transformed into mullite, a refractory material, significantly limiting the heat flow between the sample and the flame. The iron compounds contained in halloysite were also important. Despite the low concentration of iron oxides in the halloysite (in most cases it does not exceed 0.3% by mass), iron ions acted effectively as “scavenger” acceptors of free radicals that are formed in the processes of thermal degradation of the tested composites, thus increasing their thermal stability (parameter dm/dt) and fire resistance. Furthermore, the flammability of the tested composites was also reduced in the case of the straw:silica filler.

It should be clearly emphasized that for all tested fillers, the reduction in the flammability of composites containing them was the result of the reduced intensity of free radical reactions taking place in the gas phase as well as the mass and energy exchange between the sample and the flame (channel effect). In the case of the tested composites, the construction of the boundary layer was of secondary importance. In the combustion process of the tested vulcanizates, filler islands formed more often than a homogeneous, uniform boundary layer.

The flammability of a composite differs from the properties of its constituent materials. Factors such as the structure of the composite, adhesion between the fibers and the matrix, the type of polymer constituting the matrix and the type of fiber play key roles in determining the flammability of the biocomposite. There are a number of current articles and literature reviews that provide in-depth information on the state of research and solutions in the field of using mineral fillers for flame-retardant polymers, for example, on phosphorus-based flame retardants [75] and halogen-free flame retardants, including nanocomposites [76], filler minerals [77] and special flame retardants for specific polymers, such as polyolefins [78], polystyrenes [79] and polyvinylchlorides [80]. These tests, however, are carried out mainly for materials based on polypropylene, polypropylene and other commonly used materials containing natural fibers. The presented research was aimed at gaining new knowledge about the production process of elastomeric biocomposites filled with hybrid additives, as well as the phenomenal definition influencing this process.

## 4. Conclusions

The use of hybrid fillers based on mechanically modified straw allowed us to obtain a higher cross-linking density of vulcanizates, because, in addition to cross-links between the polymer chains, chemical and physical interactions with functional groups present on the fillers’ surface were created. The consequence of increasing the network node density was greater stiffness of the composition, and thus higher M_max_ and ΔM values during the vulcanization process of rubber mixtures and greater hardness of vulcanizates. The vulcanization time of the blends was slightly longer, but the obtained composites were characterized by a significantly higher tensile strength.

By analyzing the effect of the type of additive on the tested properties, it was observed that the use of silica as a modifying agent for cereal straw with the other tested functional additives resulted in lower M_min_ and ΔM during the vulcanization process, especially with higher filler content. Since the increase in torque is an indirect measure of the cross-linking density, the efficiency of the vulcanization process in silica biocomposites was probably lower and was related to the phenomenon of absorption of the components of the cross-linking unit. On the other hand, higher values of the minimum torque during cross-linking could be caused by the insufficient dispersion of fillers in the elastomer matrix. Vulcanizates containing halloysite were characterized by shorter vulcanization times and lower hardness. However, unlike the other tested cereal straw modifiers, they caused the greatest strengthening effect even at low contents (10 and 20 phr in the rubber mixture). Besides, the use of a straw-based filler with halloysite, due to good barrier properties, played an important role in limiting the speed of thermal decomposition of composites. The use of carbon black resulted in obtaining vulcanizates with the highest hardness (even 55°Sh) and good mechanical strength, which was the result of the high cross-linking density and probably the formation of its own structure in the elastomer matrix. Moreover, these vulcanizates were characterized by a satisfactory resistance to the burning process.

It is difficult to determine the most effective content of hybrid fillers, however, the results of the experiments confirm the negative influence of agglomeration on the properties of natural rubber biocomposites. The experimental rubber mixtures were prepared based on hybrid fillers, in which different weight fractions of individual components were used, i.e., straw and functional nano-additive. The analysis of the results of the conducted research suggested that increasing the weight fractions of straw in hybrid fillers resulted in the extension of the vulcanization time t_90_, an increase in the flexibility of vulcanizates and a reduction in the thermal stability of composites.

To sum up, natural rubber biocomposites containing hybrid fillers based on cereal straw powder constitute an alternative method of managing its surplus. The manufactured products were characterized by good mechanical properties and, at the same time, their cost was low due to the availability and low unit price of straw. The mechanical modification allowed us to obtain materials with specific properties, which translated into the extension of the scope of their application.

## Figures and Tables

**Table 1:** Composition of elastomer mixtures.

Sample	Straw:Nano-Additive Weight Ratio	Filler Content (phr)	NR (phr)	S (phr)	MBT (phr)	SA (phr)	ZnO (phr)
Unfilled NR		0	100	2	2	1	5
Straw:Carbon black	2:1	10	100	2	2	1	5
		20					
		30					
	5:1	10	100	2	2	1	5
		20					
		30					
Straw:Silica	2:1	10	100	2	2	1	5
		20					
		30					
	5:1	10	100	2	2	1	5
		20					
		30					
Straw:Halloysite nanotubes	2:1	10	100	2	2	1	5
		20					
		30					
	5:1	10	100	2	2	1	5
		20					
		30					

**Table 2:** Composition of hybrid biofillers.

Sample	Ratio	Straw (g)	Filler (g)
Straw:Carbon black	2:1	100	50
	5:1		20
Straw:Silica	2:1	100	50
	5:1		20
Straw:Halloysite nanotubes	2:1	100	50
	5:1		20

**Table 3:** Thermal parameters of fillers: temperature of 5% weight loss (T_5_), temperature of 50% weight loss (T_50_), the solid residue after combustion (R_700_).

Sample	T_5_ (°C)	T_50_ (°C)	R_700_ (%)
Pure Straw	146	327	13
Straw:Carbon black_2:1	205	416	30
Straw:Carbon black_5:1	200	339	19
Straw:Silica_2:1	230	452	37
Straw:Silica_5:1	222	338	25
Straw:Halloysite nanotubes_2:1	242	423	33
Straw:Halloysite nanotubes_5:1	211	342	23

**Table 4:** The rheometric parameters of the elastomer mixtures.

Sample	Straw:Nano-Additive Weight Ratio	Filler Content (phr)	M_min_ (dNm)	M_max_ (dNm)	ΔM (dNm)
Unfilled NR		0	1.01	5.89	4.88
Straw:Carbon black	2:1	10	0.72	6.26	5.54
		20	0.82	7.03	6.21
		30	0.71	7.90	7.19
	5:1	10	0.52	5.70	5.18
		20	0.76	6.53	5.77
		30	0.68	7.63	6.95
Straw:Silica	2:1	10	0.97	6.31	5.34
		20	1.01	6.94	5.93
		30	1.20	7.80	6.60
	5:1	10	0.89	6.35	5.46
		20	0.98	7.20	6.22
		30	0.31	6.89	6.58
Straw:Halloysite nanotubes	2:1	10	0.87	6.11	5.24
		20	0.91	6.77	5.86
		30	1.00	7.78	6.78
	5:1	10	0.68	6.12	5.44
		20	0.88	7.01	6.13
		30	0.93	7.94	7.01

**Table 5:** Mechanical properties of vulcanizates.

Sample	Straw:Nano-Additive Weight Ratio	Filler Content (phr)	SE_100_ (MPa)	SE_200_ (MPa)	SE_300_ (MPa)
Unfilled NR		0	0.75 ± 0.02	1.23 ± 0.04	1.73 ± 0.02
Straw:Carbon black	2:1	10	0.94 ± 0.05	1.58 ± 0.06	2.31 ± 0.02
		20	1.31 ± 0.07	2.26 ± 0.07	3.30 ± 0.04
		30	1.59 ± 0.03	2.82 ± 0.05	4.17 ± 0.06
	5:1	10	0.93 ± 0.07	1.54 ± 0.09	2.25 ± 0.04
		20	1.39 ± 0.02	2.36 ± 0.04	3.35 ± 0.03
		30	1.72 ± 0.02	2.93 ± 0.02	4.13 ± 0.02
Straw:Silica	2:1	10	0.92 ± 0.01	1.51 ± 0.05	2.23 ± 0.07
		20	1.20 ± 0.06	2.11 ± 0.02	3.07 ± 0.07
		30	1.52 ± 0.05	2.71 ± 0.03	3.90 ± 0.04
	5:1	10	1.05 ± 0.08	1.77 ± 0.11	2.53 ± 0.04
		20	1.26 ± 0.06	2.22 ± 0.01	3.18 ± 0.02
		30	1.30 ± 0.05	2.44 ± 0.08	3.47 ± 0.09
Straw:Halloysite nanotubes	2:1	10	0.96 ± 0.06	1.57 ± 0.03	2.22 ± 0.01
		20	1.19 ± 0.05	2.06 ± 0.05	2.92 ± 0.02
		30	1.48 ± 0.05	2.53 ± 0.07	3.51 ± 0.09
	5:1	10	1.00 ± 0.02	1.68 ± 0.04	2.42 ± 0.06
		20	1.32 ± 0.10	2.31 ± 0.03	3.29 ± 0.05
		30	1.54 ± 0.03	2.69 ± 0.04	3.78 ± 0.08

**Table 6:** Results of thermal analysis of biocomposites made of natural rubber.

Sample	Straw:Nano-Additive Weight Ratio	T_5_ (°C)	T_50_ (^°^C)	T_RMAX_ (^°^C)	dm/dt (%/min)	ΔT_s_ (^°^C)	P_650_ (%)
Unfilled NR		314	373	359	10.57	465–568	6.35
Straw:Carbon black	2:1	314	377	366	11.82	471–549, 549–592	8.59
		300	378	368	10.81	468–540, 540–583	8.4
		295	385	370	9.98	465–545, 545–592	8.45
	5:1	308	385	377	10.81	470–580, 550–590	7.5
		301	378	369	9.34	466–538, 538–581	8.4
		295	380	375	8.71	451–531, 531–582	8.92
Straw:Silica	2:1	310	377	368	11.51	465–552	8.37
		304	373	366	10.83	445–545	11.96
		298	379	363	10.72	438–540	10.53
	5:1	300	380	369	10.23	453–540	11.55
		311	379	371	10.99	471–555	9.16
		291	379	370	9.5	449–540	11.09
Straw:Halloysite nanotubes	2:1	314	385	370	11.67	471–565	10.1
		305	384	375	9.93	451–550	12.1
		301	374	373	9.73	445–550	16.7
	5:1	305	385	365	11.37	465–560	9.3
		300	380	376	9.76	450–550	10.4
		289	376	372	9.73	460–550	9.84

**Table 7:** Flammability results of vulcanizates analyzed on a cone calorimeter.

Sample	Straw:Nano-Additive Weight Ratio	t_i_ (s)	t_f-o_ (s)	HRR (kW/m^2^)	HRR_max_ (kW/m^2^)	tHRR_max_ (s)	THR (MJ/m^2^)	EHC (MJ/kg)	EHC_max_ (MJ/kg)	MLR (g/s)	MLR_max_ (g/s)	AMLR (g/m^2^×s)	FIGRA (kW/m^2^s)	MARHE (kW/m^2^)
Unfilled NR		60	287	264.3	458.5	155	60.2	33.4	56.9	0.07	0.265	18.21	2.95	218.3
Straw:Carbon black	2:1	78	206	309.1	550.1	145	39.4	21.3	60.7	0.12	0.314	21.67	3.79	175.9
		61	221	203.7	378.9	130	32.3	16.6	56.1	0.10	0.287	24.05	2.91	151.2
		42	215	189.1	361.7	110	32.2	16.6	74.1	0.10	0.266	19.40	3.28	158.4
	5:1	57	207	259.8	459.4	125	38.2	19.8	72.4	0.11	0.261	20.93	3.67	186.9
		50	198	214.0	378.9	120	31.8	16.7	76.7	0.11	0.293	21.93	3.15	165.5
		55	199	190.1	343.9	115	27.2	15.2	68.5	0.11	0.311	22.20	2.99	141.1
Straw:Silica	2:1	81	264	233.1	499.8	130	41.7	22.1	75.1	0.09	0.293	11.50	5.07	174.6
		103	246	237.2	452.1	135	34.2	21.7	72.6	0.09	0.278	11.70	3.34	148.7
		49	295	166.2	456.3	110	40.8	21.1	75.2	0.07	0.207	11.40	4.17	150.5
	5:1	60	229	273.2	495.2	125	46.1	22.8	59.4	0.07	0.201	11.30	3.96	167.5
		65	263	213.2	480.2	120	42.5	22.4	77.6	0.06	0.195	9.43	4.01	174.5
		54	218	210.5	406.4	115	34.5	18.6	62.9	0.05	0.182	9.45	3.53	165.5
Straw:Halloysite nanotubes	2:1	69	255	226.1	486.4	140	41.9	21.1	71.1	0.09	0.289	12.25	3.47	177.9
		45	190	238.1	449.1	110	34.9	17.8	67.9	0.11	0.523	25.80	4.08	188.6
		53	243	176.7	372.7	120	33.4	17.1	78.7	0.09	0.436	12.06	3.10	154.3
	5:1	51	208	228.9	481.4	115	35.1	18.3	72.5	0.11	0.314	22.21	4.08	179.1
		49	209	206.1	396.2	115	32.7	17.2	74.1	0.10	0.289	16.21	3.44	165.4
		55	196	213.1	373.7	125	30.5	15.4	77.4	0.12	0.291	21.27	2.98	129.6

t_i_—time to ignition, t_f-o_—time to flameout, HRR—heat release rate, HRR_max_—maximum heat release rate, tHRR_max_—time to maximum heat release rate, THR—total heat release, EHC—effective heat of combustion, EHC_max_—maximum effective heat of combustion, MLR—mass loss rate, MLR_max_—maximum mass loss rate, AMLR—average mass loss rate, FIGRA—HRR_max_/tHRR_max_, MARHE—maximum average heat release rate.

## Data Availability

Data sharing not applicable.

## References

[1] Sathishkumar T., Satheeshkumar S., Naveen J. (2014). Glass fiber-reinforced polymer composites—A review. J. Reinf. Plast. Compos..

[2] Chulawala A.M., Crasta F., Kottur V.K.N. (2020). A Review on Carbon Fibre Reinforced Polymer Composites and the Methods of Their Manufacture, Disposal and Reclamation. Lecture Notes in Mechanical Engineering.

[3] Denchev Z., Dencheva N. (2012). Manufacturing and Properties of Aramid Reinforced Composites. Synthetic Polymer-Polymer Composites.

[4] Väisänen T., Haapala A., Lappalainen R., Tomppo L. (2016). Utilization of agricultural and forest industry waste and residues in natural fiber-polymer composites: A review. Waste Manag..

[5] Verma D., Senal I. (2019). Natural fiber-reinforced polymer composites. Biomass Biopolym. Mater. Bioenergy.

[6] Al-Oqla F.M., Sapuan S.M. (2014). Natural fiber reinforced polymer composites in industrial applications: Feasibility of date palm fibers for sustainable automotive industry. J. Clean. Prod..

[7] Bassyouni M., Waheed Ul Hasan S. (2015). Biofiber Reinforcements in Composite Materials.

[8] Pickering K.L., Efendy M.G.A., Le T.M. (2016). A review of recent developments in natural fibre composites and their mechanical performance. Compos. Part A Appl. Sci. Manuf..

[9] Rajak D.K., Pagar D.D., Kumar R., Pruncu C.I. (2019). Recent progress of reinforcement materials: A comprehensive overview of composite materials. J. Mater. Res. Technol..

[10] Faruk O., Bledzki A.K., Fink H.-P., Sain M. (2014). Progress Report on Natural Fiber Reinforced Composites. Macromol. Mater. Eng..

[11] Song K. (2017). Interphase characterization in rubber nanocomposites. Progress in Rubber Nanocomposites.

[12] Kim D.Y., Park J.W., Lee D.Y., Seo K.H. (2020). Correlation between the Crosslink Characteristics and Mechanical Properties of Natural Rubber Compound via Accelerators and Reinforcement. Polymers.

[13] Faruk O., Bledzki A.K., Fink H.-P., Sain M. (2012). Biocomposites reinforced with natural fibers: 2000–2010. Prog. Polym. Sci..

[14] Ho M., Wang H., Lee J.-H., Ho C., Lau K., Leng J., Hui D. (2012). Critical factors on manufacturing processes of natural fibre composites. Compos. Part B Eng..

[15] Bourmaud A., Beaugrand J., Shah D.U., Placet V., Baley C. (2018). Towards the design of high-performance plant fibre composites. Prog. Mater. Sci..

[16] Cruz J., Fangueiro R. (2016). Surface Modification of Natural Fibers: A Review. Procedia Eng..

[17] Komuraiah A., Kumar N.S., Prasad B.D. (2014). Chemical Composition of Natural Fibers and its Influence on their Mechanical Properties. Mech. Compos. Mater..

[18] Zimniewska M., Wladyka-Przybylak M., Mankowski J. (2011). Cellulosic Bast Fibers, Their Structure and Properties Suitable for Composite Applications. Cellulose Fibers: Bio- and Nano-Polymer Composites.

[19] Marks-Bielska R., Bielski S., Novikova A., Romaneckas K. (2019). Straw Stocks as a Source of Renewable Energy. A Case Study of a District in Poland. Sustainability.

[20] Helin T., Vesterinen P., Ahola H., Niemelä K., Doublet S., Couturier C., Piotrowski S., Carus M., Tambuyser B., Hasija R. (2012). Availability of Lignocellulosic Biomass Types of Interest in the Study Regions.

[21] Passoth V., Sandgren M. (2019). Biofuel production from straw hydrolysates: Current achievements and perspectives. Appl. Microbiol. Biotechnol..

[22] Sheikh G.G., Ganai A.M., Reshi P.A., Bilal S., Mir S., Masood D. (2018). Improved paddy straw as ruminant feed: A review. Agric. Rev..

[23] Goodman B.A. (2020). Utilization of waste straw and husks from rice production: A review. J. Bioresour. Bioprod..

[24] Brown D., Shi J., Li Y. (2012). Comparison of solid-state to liquid anaerobic digestion of lignocellulosic feedstocks for biogas production. Bioresour. Technol..

[25] Talebnia F., Karakashev D., Angelidaki I. (2010). Production of bioethanol from wheat straw: An overview on pretreatment, hydrolysis and fermentation. Bioresour. Technol..

[26] Aladejana J.T., Wu Z., Fan M., Xie Y. (2020). Key advances in development of straw fibre bio-composite boards: An overview. Mater. Res. Express.

[27] Mittal V., Sinha S. (2018). Mechanical, thermal, and water absorption properties of wheat straw/bagasse-reinforced epoxy blended composites. Adv. Polym. Technol..

[28] Masłowski M., Miedzianowska J., Strzelec K. (2019). Silanized cereal straw as a novel, functional filler of natural rubber biocomposites. Cellulose.

[29] Masłowski M., Miedzianowska J., Strzelec K. (2020). The potential application of cereal straw as a bio-filler for elastomer composites. Polym. Bull..

[30] Masłowski M., Miedzianowska J., Strąkowska A., Strzelec K., Szynkowska M.I. (2018). The use of rye, oat and triticale straw as fillers of natural rubber composites. Polym. Bull..

[31] Masłowski M., Miedzianowska J., Strzelec K. (2019). Cereal straw and their physical modifications with hydrophilic and hydrophobic silica—The influence of functional hybrid material on natural rubber biocomposites. Eur. Polym. J..

[32] Panthapulakkal S., Zereshkian A., Sain M. (2006). Preparation and characterization of wheat straw fibers for reinforcing application in injection molded thermoplastic composites. Bioresour. Technol..

[33] DeArmitt C. (2017). Functional Fillers for Plastics. Applied Plastics Engineering Handbook.

[34] George J., Sreekala M.S., Thomas S. (2001). A review on interface modification and characterization of natural fiber reinforced plastic composites. Polym. Eng. Sci..

[35] Adekunle K.F. (2015). Surface Treatments of Natural Fibres—A Review: Part 1. Open J. Polym. Chem..

[36] Wang W., Sain M., Cooper P. (2006). Study of moisture absorption in natural fiber plastic composites. Compos. Sci. Technol..

[37] Puglia D., Biagiotti J., Kenny J.M. (2005). A Review on Natural Fibre-Based Composites—Part II. J. Nat. Fibers.

[38] Gurunathan T., Mohanty S., Nayak S.K. (2015). A review of the recent developments in biocomposites based on natural fibres and their application perspectives. Compos. Part A Appl. Sci. Manuf..

[39] Narayanan K.B., Suresh A.K., Sakthivel N., Thakur V.K., Thakur M.K. (2015). Eco-Friendly Polymer Nanocomposites.

[40] Kargarzadeh H., Mariano M., Huang J., Lin N., Ahmad I., Dufresne A., Thomas S. (2017). Recent developments on nanocellulose reinforced polymer nanocomposites: A review. Polymer.

[41] Fu S., Sun Z., Huang P., Li Y., Hu N. (2019). Some basic aspects of polymer nanocomposites: A critical review. Nano Mater. Sci..

[42] Ramesan M.T., Suhailath K. (2017). Role of nanoparticles on polymer composites. Micro and Nano Fibrillar Composites (MFCs and NFCs) from Polymer Blends.

[43] Balazs A.C., Emrick T., Russell T.P. (2006). Nanoparticle Polymer Composites: Where Two Small Worlds Meet. Science.

[44] Hanemann T., Szabó D.V. (2010). Polymer-Nanoparticle Composites: From Synthesis to Modern Applications. Materials.

[45] Yuan P., Tan D., Annabi-Bergaya F. (2015). Properties and applications of halloysite nanotubes: Recent research advances and future prospects. Appl. Clay Sci..

[46] Kashiwagi T., Grulke E., Hilding J., Groth K., Harris R., Butler K., Shields J., Kharchenko S., Douglas J. (2004). Thermal and flammability properties of polypropylene/carbon nanotube nanocomposites. Polymer.

[47] Spahr M.E., Rothon R. (2016). Carbon Black as a Polymer Filler. Polymers and Polymeric Composites: A Reference Series.

[48] Mwila J., Miraftab M., Horrocks A.R. (1994). Effect of carbon black on the oxidation of polyolefins—An overview. Polym. Degrad. Stab..

[49] Janowska G., Rybiński P. (2008). Influence of carbon black on thermal properties and flammability of cross-linked elastomers. J. Therm. Anal. Calorim..

[50] Krauskopf K.B., Loague K. (2003). Environmental Geochemistry. Encyclopedia of Physical Science and Technology.

[51] Belyakov V.N., Belyakova L.A., Varvarin A.M., Khora O.V., Vasilyuk S.L., Kazdobin K.A., Maltseva T.V., Kotvitskyy A.G., Danil de Namor A.F. (2005). Supramolecular structures on silica surfaces and their adsorptive properties. J. Colloid Interface Sci..

[52] Lee D.W., Yoo B.R. (2016). Advanced silica/polymer composites: Materials and applications. J. Ind. Eng. Chem..

[53] Hewitt N. (2007). Silica as a reinforcing filler. Compounding Precipitated Silica in Elastomers.

[54] Zou H., Wu S., Shen J. (2008). Polymer/Silica Nanocomposites: Preparation, Characterization, Properties, and Applications. Chem. Rev..

[55] Maciejewska M., Siwek M. (2020). The Influence of Curing Systems on the Cure Characteristics and Physical Properties of Styrene–Butadiene Elastomer. Materials.

[56] Flory P.J., Rehner J. (1943). Statistical Mechanics of Cross-Linked Polymer Networks I. Rubberlike Elasticity. J. Chem. Phys..

[57] Roovers J., Toporowski P.M. (1990). Characteristic Ratio and Plateau Modulus of 1,2-Polybutadiene. A Comparison with Other Rubbers. Rubber Chem. Technol..

[58] Ahmed K., Nizami S.S., Raza N.Z., Habib F. (2013). The effect of silica on the properties of marble sludge filled hybrid natural rubber composites. J. King Saud Univ. Sci..

[59] ISO 37:2017 (2017). Rubber, Vulcanized or Thermoplastic—Determination of Tensile Stress-Strain Properties.

[60] ISO 48-4:2018 (2018). Rubber, Vulcanized or Thermoplastic—Determina-Tion of Hardness—Part 4: Indentation Hardness by Durometer Method (Shore Hard-Ness).

[61] Pegoretti A., Dorigato A., Brugnara M., Penati A. (2008). Contact angle measurements as a tool to investigate the filler–matrix interactions in polyurethane–clay nanocomposites from blocked prepolymer. Eur. Polym. J..

[62] Christy A.A. (2014). The Nature of Silanol Groups on the Surfaces of Silica, Modified Silica and Some Silica Based Materials. Adv. Mater. Res..

[63] Szpilska K., Czaja K., Kudla S. (2015). Halloysite nanotubes as polyolefin fillers. Polimery.

[64] Yu H., Liu R., Shen D., Wu Z., Huang Y. (2008). Arrangement of cellulose microfibrils in the wheat straw cell wall. Carbohydr. Polym..

[65] Asim M., Paridah M.T., Chandrasekar M., Shahroze R.M., Jawaid M., Nasir M., Siakeng R. (2020). Thermal stability of natural fibers and their polymer composites. Iran. Polym. J..

[66] Zhang A., Zhang Y., Zhu Z. (2019). Thermal properties of Halloysite nanotubes (HNTs) intercalation complexes-A review. ChinaBiofilms.

[67] Yuan P., Tan D., Aannabi-Bergaya F., Yan W., Fan M., Liu D., He H. (2012). Changes in Structure, Morphology, Porosity, and Surface Activity of Mesoporous Halloysite Nanotubes under Heating. Clays Clay Miner..

[68] Kosmalska A., Zaborski M., Ślusarski L. (2003). Adsorption of curatives and activity of silica toward elastomers. Macromol. Symp..

[69] Fröhlich J., Niedermeier W., Luginsland H.D. (2005). The effect of filler-filler and filler-elastomer interaction on rubber reinforcement. Compos. Part A Appl. Sci. Manuf..

[70] Sadiku E.R., Babul Reddy A., Gnanasekarana D., Oboirien B., Aderibigbe B.A., Varaprasad K., Goddeti S.M.R. (2016). Nanostructured Polymer Blends for Gas/Vapor Barrier and Dielectric Applications. Design and Applications of Nanostructured Polymer Blends and Nanocomposite Systems.

[71] Cui Y., Kumar S., Rao Kona B., van Houcke D. (2015). Gas barrier properties of polymer/clay nanocomposites. RSC Adv..

[72] Rybiński P., Janowska G. (2013). Flammability and other properties of elastomeric materials and nanomaterials. Part I. Nanocomposites of elastomers with montmorillonite or halloysite. Polimery.

[73] Mngomezulu M.E., John M.J., Jacobs V., Luyt A.S. (2014). Review on flammability of biofibres and biocomposites. Carbohydr. Polym..

[74] Rybiński P., Syrek B., Masłowski M., Miedzianowska J., Strzelec K., Żukowski W., Bradło D. (2018). Influence of Lignocellulose Fillers on Properties Natural Rubber Composites. J. Polym. Environ..

[75] Levchik S.V., Weil E.D. (2006). A Review of Recent Progress in Phosphorus-based Flame Retardants. J. Fire Sci..

[76] Lu S.-Y., Hamerton I. (2002). Recent developments in the chemistry of halogen-free flame retardant polymers. Prog. Polym. Sci..

[77] Bourbigot S., Duquesne S. (2007). Fire retardant polymers: Recent developments and opportunities. J. Mater. Chem..

[78] Weil E.D., Levchik S.V. (2008). Flame Retardants in Commercial Use or Development for Polyolefins. J. Fire Sci..

[79] Weil E.D., Levchik S.V. (2007). Flame Retardants for Polystyrenes in Commercial Use or Development. J. Fire Sci..

[80] Weil E.D., Levchik S., Moy P. (2006). Flame and Smoke Retardants in Vinyl Chloride Polymers—Commercial Usage and Current Developments. J. Fire Sci..

